# All‐Optical Diffractive Operators for Rapid, Computer‐Free Morphological Transformations

**DOI:** 10.1002/nap2.70031

**Published:** 2026-02-22

**Authors:** Yuxiang Sun, Fenglei Wang, Jing Han, Geyang Qu, Zezheng Zhang, Yan Wei, Chuang Yang, Qifeng Ruan, Shengjie Wang, Heming Wei, Chaoran Huang, Jun Guan, Jingtian Hu

**Affiliations:** ^1^ Ministry of Industry and Information Technology Key Lab of Micro‐Nano Optoelectronic Information System Guangdong Provincial Key Laboratory of Semiconductor Optoelectronic Materials and Intelligent Photonic Systems Harbin Institute of Technology Shenzhen China; ^2^ School of Science and Engineering The Chinese University of Hong Kong (Shenzhen) Shenzhen Guangdong China; ^3^ Shanghai University Shanghai China; ^4^ New York University Shanghai Shanghai China; ^5^ Department of Electronic Engineering The Chinese University of Hong Kong Hong Kong China; ^6^ Quantum Science Center of Guangdong‐Hong Kong‐Macao Greater Bay Area Shenzhen China; ^7^ Key Laboratory of Photonic Technology for Integrated Sensing and Communication Ministry of Education Guangdong University of Technology Guangzhou China

**Keywords:** diffractive networks, machine learning, morphological transformations

## Abstract

Morphological transformations are playing a key role in visual information processing with diverse applications ranging from bioimaging to video surveillance and environmental monitoring. However, these operations are becoming increasingly computationally intensive, requiring substantial memory and processing power as the size of image datasets expands. This paper describes a fast, highly parallel approach to perform morphological transformations by diffractive computing. These all‐optical processors consist of successive diffractive surfaces designed to perform dilation and erosion operations by learning the relations between input and transformed images via a deep learning‐based optimization process. Unlike existing digital methods, our free‐space diffractive devices implement these transformations in a computer‐free manner by directly processing the optical wavefront. The cascaded diffractive architecture further enables image denoising and flexible tuning of the extent and directionality of erosion/dilation through the same training process by adjusting target image datasets, realizing the synthesis of diverse transformation kernels on demand. We also demonstrate that the optical process is scalable and can process large volumes of visual information in a highly parallel manner. Experimentally, we realize such a diffractive network in a reflection configuration using a phase‐only spatial light modulator (SLM) and perform morphological transformations on both amplitude‐ and phase‐encoded images.

## Graphical abstract

This paper shows an all‐optical, computer‐free approach to realize morphological transformations on visual data. Free‐space optical devices consisting of successive diffractive layers are trained through a deep learning‐based optimization process to perform erosion and dilation for images with diverse styles. The optical computing system is scalable for processing large images with near‐zero latency and demonstrates applications in image denoising.

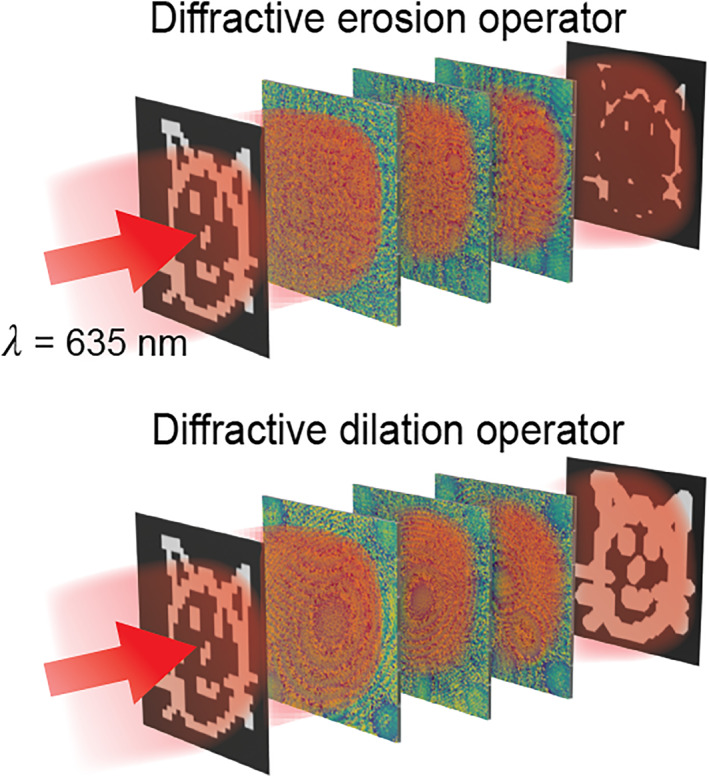

## 1 Introduction

Morphological operations [1, 2] are crucial algorithmic tools for computer vision technologies ranging from materials/device defect detection [3] and medical diagnosis [4, 5] to security surveillance [6, 7] and geospatial analysis [8, 9]. These operations constitute a family of nonlinear transformations that reshape object structures through predefined kernels [10]. The most representative examples include erosion and dilation, which can be further combined into composite forms such as opening and closing. More advanced reconstruction filters are also employed for edge extraction [11] and feature enhancement [12]. These transformations are also essential enablers of vision‐based functionalities in artificial intelligence [5, 7] because of their prevalent use in feature extraction [8], noise reduction [13], and neural network implementation [14]. Although simple in mathematical expression, morphological transformations can be computationally expensive [1, 2, 6] on a global scale with the explosive growth of data‐intensive technologies [15], such as autonomous driving and the internet of things [16], which require real‐time processing of massive images and video streams. Although GPU‐based computing can accelerate these operations significantly [17], their fast computation for large images on portable platforms [17] still remains a challenge but is critical for the development of futuristic intelligent edge devices.

Free‐space optical computing platforms are powerful processors for efficient processing and computation using visual information because of their advantages in compactness, speed, and parallelism [18, 19, 20, 21]. For example, metasurfaces [22, 23, 24] consisting of structured subwavelength units can perform computations such as convolution [25, 26], integration [27], and differentiation [28] over the wavefront at a speed limited only by light propagation. Diffractive optical networks consisting of multiple wavefront processing layers further enable sophisticated functionalities ranging from machine learning and inference [18] to linear transformations [29, 30] by merging the Huygens–Fresnel principle with deep learning‐based training processes. All‐optical implementations of morphological operations, which are typically nonlinear [4, 17], have not yet been demonstrated, primarily because free‐space diffractive systems are linear in nature and can hardly realize nonlinear mappings directly. Nevertheless, such processors hold significant promise for on‐the‐fly image processing in compact and portable optical systems.

Here, we demonstrate a class of morphological diffractive operators that perform on‐the‐fly dilation and erosion operations on incoming visual data by diffractive computing. These optical networks were obtained in a deep learning‐based training process, where the phase‐delay values of the diffractive units were optimized by learning the relationship between input and transformed images from a massive number of examples. Numerical blind‐testing results verify that the trained diffractive networks can perform dilation and erosion on new, unseen images of diverse styles, ranging from handwritten digits and letters to customized line patterns. Composite forms of such diffractive processing modules further realize effective image denoising through a combination of erosion and dilation. Importantly, these passive optical networks can be trained to perform both dilation and erosion of images with controllable scale and directionality to extract image features with diverse length scales. We further demonstrate that our free‐space approach is scalable for processing large images up to 640 × 640 pixels. We experimentally verify the proposed diffractive network in a reflective configuration for performing morphological transformations on phase‐ and amplitude‐encoded image information. The system, built with a phase‐only SLM and mirror‐assisted multiple diffraction, successfully executes erosion and dilation operations in real time, with a processing speed limited only by the light source intensity and image sensor sensitivity. Overall, this compact, purely optical platform provides a practical route toward scalable, task‐specific morphological processing, with broad applications in next‐generation optical artificial intelligence and computer vision hardware.

## 2 Results and Discussion

### 2.1 Overview of All‐Optical Morphological Diffractive Operators

**FIGURE 1:**
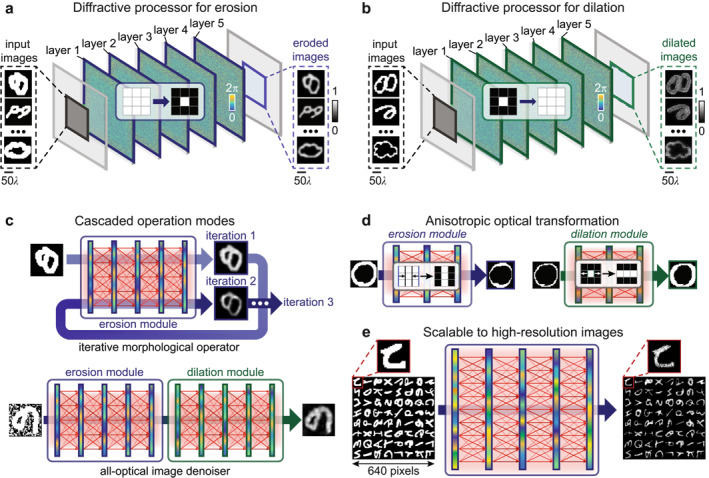
Free‐space optical networks for versatile morphological transformations. Schemes showing our designs of free‐space diffractive networks for (a) erosion and (b) dilation of images. The all‐optical network performs an isotropic erosion/dilation on the input images with a single pass of light propagation and obtains the eroded output images at the detector. (c) Cascaded configurations of multiple diffractive networks enable advanced image processing tasks, including multistage erosion/dilation and morphological denoising. (d) The diffractive networks were also capable of anisotropic morphological transformations for the extraction and enhancement of directional features in images. (e) Scalability demonstration of the diffractive morphological operators for the parallel processing of large, high‐resolution images (up to 640 × 640 pixels). Copyright (Quick, Draw! samples): Example doodles shown in this figure are from the Quick, Draw! dataset (Ref. [33]), Google LLC, used under the Creative Commons Attribution 4.0 International (CC BY 4.0) license with attribution. The samples were preprocessed (e.g., binarization/cropping/resizing) for this work. Example characters shown in this figure are from the EMNIST dataset (Ref. [34]), distributed via NIST and derived from NIST Special Database 19. The samples were used for research and were preprocessed (e.g., binarization/cropping/resizing) for this work.

Our free‐space optical networks for morphological transformations consisted of multiple diffractive surfaces [31, 32] that were designed via a deep learning‐based training process by learning from pairs of raw and target transformed images. Figure 1a,b depicts typical designs of diffractive networks for two common morphological transformations, that is, erosion and dilation, respectively. The diffractive networks performed the transformation of input images represented by the 2D optical intensity/phase profiles by the alternating processes of (1) free‐space propagation of the wavefront from a previous diffractive layer (or input plane) to the next layer (or output plane) and (2) wavefront modulation by diffractive layers. Therefore, the input wavefront transmitting through the device was modulated by consecutive diffractive layers in the process to generate the transformed (i.e., dilated and eroded) images at the output plane. For such free‐space optical networks to generate the desired output images, the diffractive layers, which were restricted to modulate wavefront phases in this study, were optimized in a deep learning‐based training process of a diffractive network. To start the training, the phase modulation coefficients on each layer were first initialized to random values. In each epoch of the training, a large number of images were input into the randomly initialized diffractive network whose outputs were compared with the corresponding transformed images by the desired morphological transformation to calculate losses. Then, the diffractive layer designs were adjusted via the backpropagation of such losses for each batch of input images. A typical training process for this study required ∼200 epochs. After training, the entire image processing task was performed in a single‐shot manner, where all computations were finished by a one‐time propagation of the input light profiles through the diffractive layers, enabling extraordinary processing speed and parallelism. In addition, practical image processing tasks (such as edge detection [35, 36], noise removal [37], and feature enhancement [38]) often require a combination of morphological transformations that are sequentially applied in a pipeline. We verified the feasibility of our diffractive image processing modules in several such tasks, including the opening/closing of image features by repeated optical erosion/dilation and image denoising by an erosion operation followed by a dilation operation (Figure 1c).

In addition, the deep learning‐based design process allowed the scaling of the structuring elements to adjust the extent of dilation/erosion operations to process image features at different length scales. This adjustment to our diffractive design was realized simply by adjusting the datasets of transformed images that serve as the target ground‐truth images for the network output. The same strategy also allowed the free‐space network to perform anisotropic image processing to effectively capture anisotropic geometrical structures [39] in visual information, facilitating complex image processing tasks such as edge detection [40] and image enhancement [41]. As shown by Figure 1d, our diffractive networks allowed directional dilation and erosion processes based on anisotropic structuring elements, enabling varying directions of morphological changes, such as erosion or dilation restricted to the horizontal/vertical direction. Because implementations of erosion/dilation algorithms on digital computers exhibit an O(*n* ^2^) time complexity for an image consisting of n×n pixels [42], the execution of these operations on electronic platforms becomes inefficient as the size of input images grows [43]. In contrast, our diffractive networks represented an inherently parallel platform that completed image filtering tasks through a single pass of light propagation, demonstrating substantial advantages in processing speed for large‐sized images, though system performance might be constrained by numerical aperture limitations and edge information fidelity for extremely large image formats. To demonstrate this potential, we tested the scalability of our all‐optical networks from small images of 80 × 80 up to large images of 640 × 640 pixels and confirmed the consistency in our device performance for all image sizes (Figure 1e).

### 2.2 Diffractive Morphological Operators for Isotropic Transformations

**FIGURE 2:**
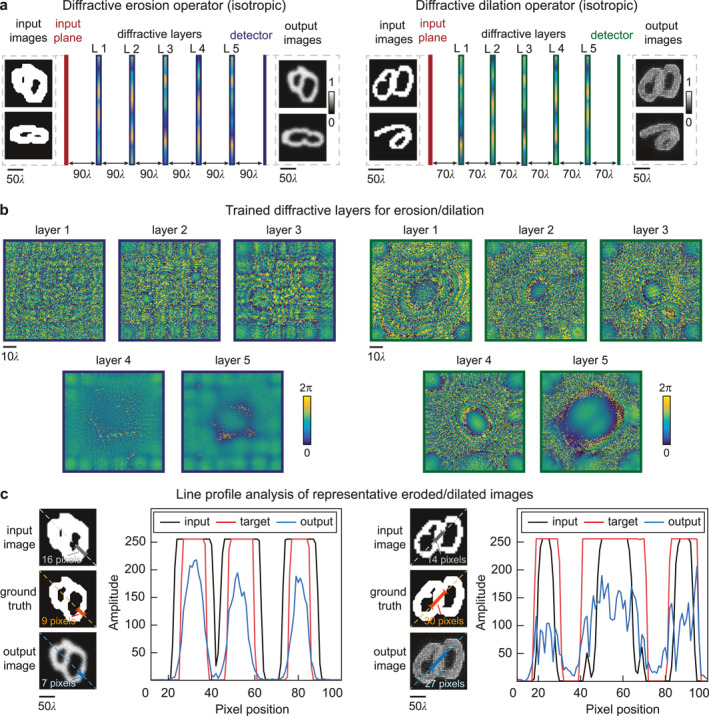
Design and performance analysis of all‐optical diffractive networks for morphological transformations. (a) Schemes showing the architecture of our 5‐layer diffractive networks for performing erosion (left) and dilation (right). (b) The optimized designs of the phase‐modulating diffractive layers obtained by the deep learning‐based training process for erosion (left) and dilation (right). (c) Line profile analysis performed for representative test images before and after morphological transformation. The test images used in Panel (c) were never exposed to the diffractive networks during the training process. The pixel distribution of the input, output, and target images was quantitatively compared in a side‐by‐side illustration to demonstrate the effectiveness of our diffractive networks. Copyright (Quick, Draw! samples): Example doodles shown in this figure are from the Quick, Draw! dataset (Ref. [33]), Google LLC, used under the Creative Commons Attribution 4.0 International (CC BY 4.0) license with attribution. The samples were preprocessed (e.g., binarization/cropping/resizing) for this work. Example characters shown in this figure are from the EMNIST dataset (Ref. [34]), distributed via NIST and derived from NIST Special Database 19. The samples were used for research and were preprocessed (e.g., binarization/cropping/resizing) for this work.

We used wave optics principles to establish the numerical model of the diffractive network for performing all‐optical image transformations. Figure 2a presents the designs of our diffractive network for isotropic erosion and dilation of binary images. For both operations, the network consisted of 5 layers of spatially structured diffractive surfaces. Although the overall design could be scaled to all wavelengths [30], our device specifically targeted operation in the visible spectrum at a wavelength of *λ* = 635 nm. We started with small‐scale image inputs with 80 × 80 pixels represented in an optical field, where each pixel corresponded to a physical size of 0.6 μm. Each diffractive layer consisted of 160 × 160 phase modulation units, each with a lateral size of ∼ 0.47λ and a trainable phase modulation coefficient in the range ϕ = 0–2π. The phase modulation elements were optimized using a supervised deep learning framework that learned the complex‐valued optical mapping between input and target intensity patterns. To ensure robust generalization across diverse structural patterns, the model was trained and evaluated using a diversified dataset composed of a subset of the EMNIST dataset [34], selected categories from the QuickDraw dataset [33], and customized grating images with varied widths and orientations (see Section 4 for details). Once trained, the diffractive surfaces with optimized network parameters could perform real‐time, passive, and highly parallel optical processing as the wavefront carrying the input image propagated through the system. For these designs, the layer‐to‐layer spacing between diffractive surfaces, together with the distances from the input plane to the first layer and from the final layer to the output plane, were set to 90λ and 70λ for the erosion and dilation tasks, respectively, to achieve an optimal trade‐off between the effective receptive field [18] and the numerical aperture [44] of the system (Supporting Information S1: Figure S1). Analysis further confirmed that the selected size of the diffractive network (i.e., 5 layers, 160 × 160 neurons) achieved optimal performance, and the training became increasingly challenging for larger model sizes (Supporting Information S1: Figures S2 and S3).

The abovementioned numerical model served as the forward model that enabled the deep learning‐based optimization process for our device. To perform such a training process, we also adopted loss functions based on mean squared error (MSE) [45] and Pearson correlation coefficient (PCC) [46] to quantify the errors of the diffractive networks by comparing their processed images with the desired outputs based on image morphological processing tasks (see Section 4 for details). Figure 2b shows the designs of the phase‐modulating diffractive layers optimized for erosion (left) and dilation (right) tasks, respectively, with backpropagation of losses (see Section 4 for details). For both designs, the minimum feature size of each modulation unit was set to 0.47λ, and the phase modulation range spanned from 0 to 2π.

Figure 2c illustrates representative testing results for the diffractive morphological operators for isotropic erosion and dilation tasks, respectively. A pair of previously unseen test images—one for erosion and one for dilation—was selected to visualize how the diffractive system modulated spatial structures. As shown in Figure 2c, the trained diffractive networks successfully reduced/expanded the edge thickness according to the intended erosion/dilation transformation, respectively. For the erosion task, in particular, the contour width extracted from the line profiles decreased from 16 pixels to 7 pixels, closely matching the 9‐pixel width of the expected ground truth. We further tested the generalization capability of the model using a diverse dataset of 36,000 test images, including EMNIST digits, QuickDraw sketches, and synthetic grating patterns, and performed a statistical analysis on the linewidth reduction caused by the optical erosion process on the grating dataset (see Section 4 for details). For the grating dataset with varying widths and orientations, cross‐sectional profiles perpendicular to the grating direction were measured to compute the average change in linewidth before and after processing by the diffractive networks. The results revealed a mean erosion magnitude of approximately 8 pixels, consistent with the designed structuring element, confirming that the diffractive networks maintained reliable morphological transformation capabilities across diverse and unseen image types. Specifically, for horizontal gratings (*θ* = 0°), the average linewidth reduction was 3.72 pixels, closely matching the target reduction of 3.65 pixels, with a standard deviation of 1.63 pixels. For vertical gratings (*θ* = 90°), the average reduction was 3.73 pixels, also closely aligned with the target value of 3.67 pixels, with a standard deviation of 1.86 pixels. These results further validated the network's ability to perform consistent and accurate morphological transformations across multiple orientations. Detailed measurement results are provided in Supporting Information S1: Figure S4, which illustrates the statistical consistency of the erosion effect across different grating directions. The slight over‐shrinkage was likely attributed to the inherent limitations of diffraction‐based analog optical computation when approximating the transformations to generate sharp step functions [47]. Despite this discrepancy, the processed images overall achieved an SSIM of up to 0.897 compared to the target images when tested using the test dataset, demonstrating strong generalization across both natural and synthetic image domains.

For the dilation task, we employed a quantitative evaluation approach similar to that used in the erosion analysis. A diverse set of test images, including EMNIST digits, QuickDraw sketches, and synthetic grating patterns, was used to assess the generalization capability of the diffractive networks. As shown in Figure 2c, for a typical input grating, line profiles were extracted perpendicular to the grating direction to measure the edge width before and after processing. As a representative example, the contour width of a single grating increased from 10 pixels in the input to 23 pixels after processing, compared to the target width of 30 pixels. As shown in Supporting Information S1: Figure S5, for the grating dataset with varying widths and orientations, the average linewidth expansion was quantified. Specifically, for horizontal gratings (*θ* = 0°), the mean width increase was 3.65 pixels, with a standard deviation of 1.52 pixels; for vertical gratings (*θ* = 90°), the mean expansion was 3.31 pixels, with a standard deviation of 1.38 pixels. These results confirm that the diffractive networks can perform consistent and orientation‐independent dilation across diverse and unseen inputs.

The slightly lower‐than‐expected expansion is likely associated with the physical limitations of the compact diffractive architecture, which constrains the effective propagation distance and the spatial extent over which constructive interference can simulate larger kernels. Despite this, the processed images achieved an SSIM of up to 0.803 in the EMNIST dataset when compared with the target images, demonstrating strong generalization across both natural and synthetic datasets.

### 2.3 Cascaded Diffractive Networks for Morphological Image Processing

**FIGURE 3:**
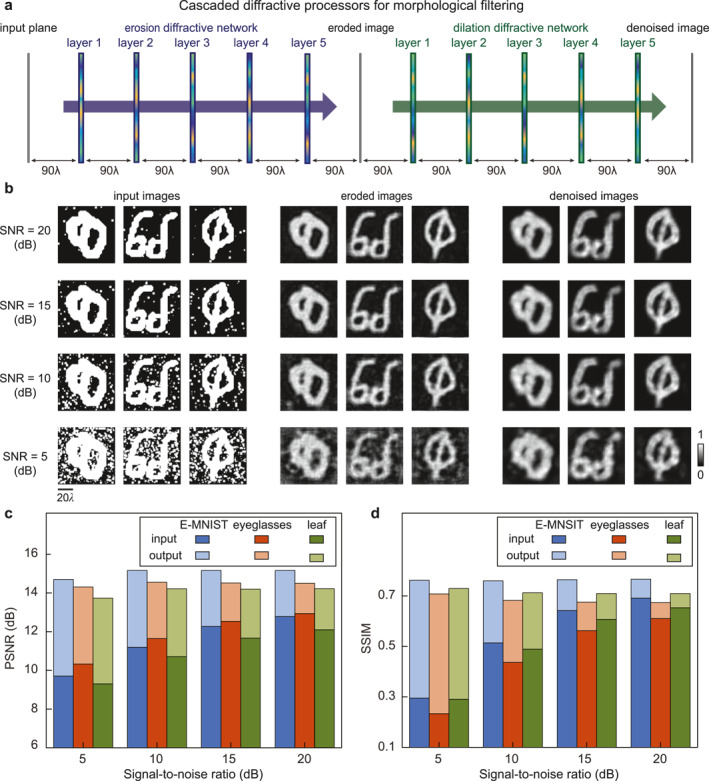
Composite morphological diffractive operators for free‐space all‐optical image denoising. (a) Cascaded diffractive operators for morphological opening built from separately optimized erosion and dilation diffractive networks. (b) Input images with varying levels of noise (SNR = 20, 15, 10, and 5 dB) from the EMNIST, eyeglasses, and leaf datasets, and the corresponding eroded and denoised images after applying the all‐optical morphological opening operation. (c) Peak signal‐to‐noise ratio (PSNR) values for the EMNIST, eyeglasses, and leaf datasets before and after denoising. (d) Structural similarity index (SSIM) values for the same datasets before and after denoising. Copyright (Quick, Draw! samples): Example doodles shown in this figure are from the Quick, Draw! dataset (Ref. [33]), Google LLC, used under the Creative Commons Attribution 4.0 International (CC BY 4.0) license with attribution. The samples were preprocessed (e.g., binarization/cropping/resizing) for this work. Example characters shown in this figure are from the EMNIST dataset (Ref. [34]), distributed via NIST and derived from NIST Special Database 19. The samples were used for research and were preprocessed (e.g., binarization/cropping/resizing) for this work.

Existing image processing pipelines often involve sequences of morphological transformations where dilation and erosion are essential building blocks. To illustrate the ability of our diffractive networks to play a role in such pipelines, we further illustrated several task‐specific designs constructed by cascading multiple diffractive networks in all‐optical frameworks. Figure 3a shows our all‐optical approach to implement composite morphological operations by cascading two separately optimized diffractive networks, one for erosion and the other for dilation. For such opening/denoising demonstration, the erosion network was optimized with a 5 × 5 all‐1 structuring element to perform a single erosion on the input image, whereas the dilation network was optimized with the same 5 × 5 structuring element to perform dilation. This symmetric opening operation removes small bright noise points in the input image, providing morphological denoising capabilities. The detailed phase distributions of all diffractive layers are presented in Supporting Information S1: Figure S6.

For numerical testing of our composite diffractive denoiser, we used images with added salt noise, and the output images processed by the digital morphological opening operations (i.e., erosion + dilation) were used as the ground truth. For this blind test, we used images from the EMNIST and QuickDraw (eyeglasses and leaf) datasets that were never presented to the networks during training with additional noise levels where the signal‐to‐noise ratios (SNR) ranged from 20 to 5 dB. These noisy inputs were then optically processed by the composite diffractive morphological models that performed erosion followed by dilation. As shown in Figure 3b, after the all‐optical opening operation, the noise points in the input image were effectively removed for all these noise levels, demonstrating the effectiveness of our diffractive networks for denoising tasks. To provide a quantitative analysis of denoising efficacy, we further compared the peak SNR (PSNR) values for the images before and after optical denoising by our diffractive networks. Figure 3c shows the PSNR values evaluated for the EMNIST, eyeglasses, and leaf datasets at different noise levels as the baseline and the metric evaluated for the denoised images. This comparative study showed that, although the noisy images with SNR = 5–20 dB exhibited drastically different PSNR values ranging from 9.3 to 12.9, they consistently showed significantly improved PSNR values around 14 after denoising. This comparative study showed that the free‐space optical denoiser based on composite diffractive networks could consistently recover information from noisy images down to an SNR of 5 dB. Figure 3d further shows the SSIM values of the noisy and denoised images using the corresponding clean, noise‐free images as the ground truth (SSIM = 1). As shown by Figure 3d, the increasing noise levels from SNR = 20 to 5 dB significantly degraded the SSIM of the image from ∼0.65 to ∼0.25 for the three test datasets. Remarkably, the denoised images for inputs at all noise levels exhibited a recovered SSIM of approximately 0.7, which indicates a decent similarity to the original noise‐free image.

To further evaluate the external generalizability of the diffractive denoising network to real‐world industrial scenarios, we additionally tested the trained model by processing a dataset of surface defects. Specifically, 200 images were randomly selected from the magnetic tile surface defects dataset [48] and were used solely for testing without any modification or retraining of the diffractive networks. The same cascaded erosion–dilation configuration was applied to suppress noise patterns commonly encountered during defect formation and imaging processes. Representative results are provided in Supporting Information S1: Figure S7, which shows that the proposed all‐optical diffractive denoiser maintained effective noise suppression performance on these real‐world images, thereby demonstrating its robustness and generalization beyond synthetic symbol datasets. We also verified that the cascaded architecture was robust against misalignments (Δ*x*) between the numerical models of the erosion and dilation networks (Supporting Information S1: Figure S8).

### 2.4 Morphological Diffractive Operators With Varying Structuring Elements

The ability to control the size and shape of the structuring element is essential for many computer vision tasks, including edge detection [49] and image denoising [50]. Smaller structuring elements (e.g., $S_{3\times 3}$) are generally used for fine‐grained texture preservation or subtle boundary refinement, whereas larger elements (e.g., $S_{5\times 5}$, $S_{7\times 7}$, and $S_{9\times 9}$) are more effective for removing noise or extracting coarse features in highly cluttered images [51]. Here, $S_{n\times n}$ denotes a square structuring element of size n×n, where the values are set to all ones to represent standard isotropic morphological operations. Although light propagation in free space naturally exhibits erosion‐like transformations on image features, such effects are inherently uncontrolled and nondeterministic (Supporting Information S1: Figure S9). To realize such tuning of structuring elements in a controllable manner, we optimized the morphological transformation corresponding to each individual structuring element from $S_{3\times 3}$ to $S_{9\times 9}$ (Supporting Information S1: Figure S10). For erosion, adjustment of the kernel from $S_{3\times 3}$ to $S_{5\times 5}$ resulted in average linewidth reductions of ∼1.5 pixels with an SSIM of 0.794. When enlarged to $S_{9\times 9}$ and beyond, the average erosion magnitude plateaued at approximately 8.16 pixels and 8.65 pixels for horizontal and vertical gratings, respectively, with SSIM dropping to around 0.721. For the dilation task, similar quantitative analysis showed that increasing the kernel to $S_{5\times 5}$ produced average linewidth expansions of ∼3.6 pixels with an SSIM of 0.618. When the kernel was further increased to $S_{9\times 9}$ and above, the average dilation plateaued at approximately 6.79 pixels, and SSIM decreased to about 0.362.

These results indicated that morphological transformations with extremely large structuring elements might exceed the processing capabilities of our compact optical design. The performance degradation observed with larger structuring elements (e.g., $S_{5\times 5}$ to $S_{9\times 9}$) indicates that the current compact diffractive design cannot faithfully realize very long‐range morphological interactions. As the structuring element grows, the desired transformation becomes more global and strongly nonlinear [52], whereas our architecture remains a finite‐depth, linear optical system with a limited number of trainable phase parameters. This mismatch between task complexity and model capacity leads to saturation of the achievable erosion/dilation magnitude and a drop in SSIM for very large kernels. These observations suggest a practical upper bound on the structuring element size that can be reliably implemented with the present diffractive architecture and motivate future work on architectures with increased depth, effective receptive field, or auxiliary nonlinear stages to better support large‐kernel morphological operations. These findings highlighted the fundamental trade‐off between transformation fidelity and the complexity of the task while also emphasizing the task‐specific importance of structuring element scaling in morphological image analysis.

To address this limitation, we introduced an alternative strategy that achieves high‐fidelity erosion and dilation with large effective structuring elements through iterative application of small‐kernel diffractive networks. Such recursive composition has been well established in conventional digital morphology [53] and can be physically realized by cascading multiple optical modules. A diffractive network was trained to perform an $S_{3\times 3}$ all‐ones structuring element with an average reduction of 1.15 pixels per cycle and an SSIM value of > 0.8 (Supporting Information S1: Figure S11). Then, we demonstrated the erosion of images to various extents by cyclic application of this diffractive network, where the optically eroded outputs were input into the network again for further transformation. For the grating test set, the mean erosion widths for 2, 3, and 4 rounds of diffractive erosions were 3.53, 8.93, and 12.53 pixels, respectively, and the processed images exhibited significantly improved sharpness and fidelity. The slight degradation in image quality with increasing cascade rounds mainly arises from cumulative error propagation across non‐reoptimized stages; in principle, the cascaded performance could be further improved by applying cascade‐aware reoptimization, for example, stagewise fine‐tuning or end‐to‐end joint optimization of the diffractive networks [54]. Besides isotropic morphological transformations, directional erosion and dilation operations can also play a key role in edge enhancement [55] and image denoising [56], which require selective extraction of features with certain orientations. In this work, we further extend our diffractive networks to directional morphological transformations using anisotropic structuring elements such as $S_{1\times 3}$ and $S_{3\times 1}$ kernels. As shown in Supporting Information S1: Figure S12, these diffractive networks effectively extract orientation‐specific features, achieving average SSIMs of 0.803 and 0.61 for erosion and dilation tasks, respectively, across EMNIST and QuickDraw test sets. These observations confirmed that cyclic application of the same morphological diffractive network could emulate morphological transformations of varying strength, often with better accuracy and stability than direct complex‐kernel training. This property further supported the view that our morphological diffractive networks can serve as a modular optical computing primitive, from which arbitrary structuring element shapes and sizes can be composed through appropriate cascading.

### 2.5 Scalability of the Diffractive Network for Processing Large, High‐Resolution Images

One increasingly important issue for computer vision is that the computation of transformations often becomes costly as the input images increase in size. This issue is particularly critical recently because visual data are being produced at unprecedented rates [57], creating a challenge for fast processing. In our initial designs (see Section 2), we focused on processing binary images of size 80 × 80 pixels, which served as a representative benchmark for compact diffractive network configurations. To demonstrate that our approach also scales well to higher‐resolution data, we developed several enlarged versions of our morphological diffractive networks capable of handling large input images. Notably, despite its simplicity, the complexity of dilation/erosions on digital platforms (defined by a K×K structuring element) is $O\left(H\times W\times K^{2}\right)$, where *H* and *W* are the size of the input image in terms of pixels. In other words, conventional algorithms for these operations require *n* ^2^ times more steps to complete when the images are enlarged by a factor of n in both dimensions, making the process slow for high‐resolution images. In contrast, our diffractive networks provided a highly scalable approach for ultrafast visual information processing because of their inherent advantages in speed and parallelism that can significantly reduce the computational overhead of image processing.

**FIGURE 4:**
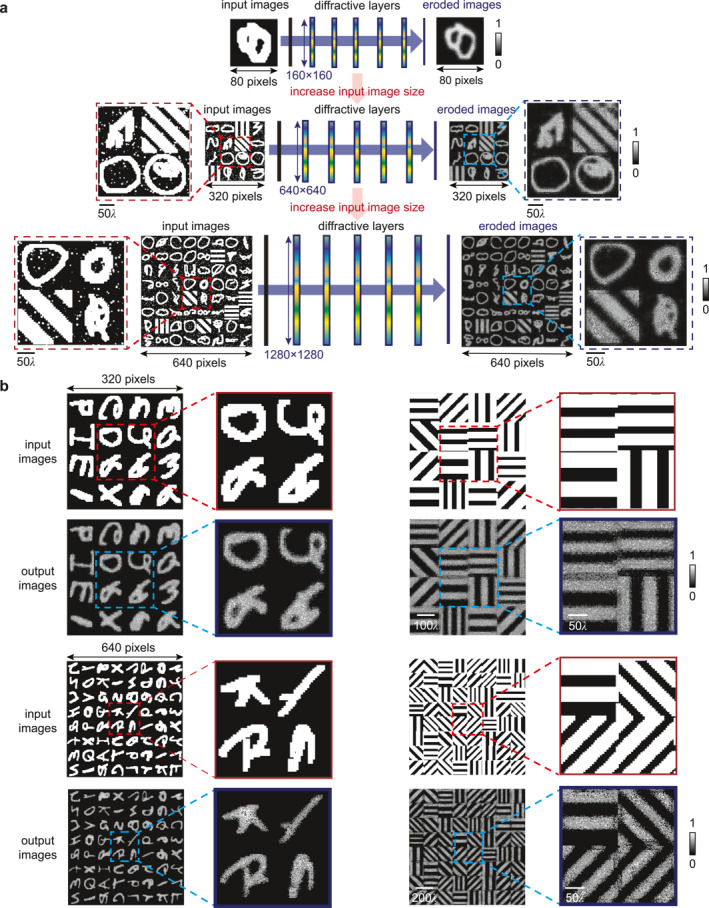
Scalable morphological processing of large images by diffractive networks. (a) Schemes showing the architectures of our diffractive networks with expanded layers (up to 1280 × 1280) for processing scaled‐up images up to 640 × 640 pixels. (b) Representative test results from the diffractive optical erosion operator using large images by stitching 4 × 4 and 8 × 8 EMNIST letters and gratings. Copyright (Quick, Draw! samples): Example doodles shown in this figure are from the Quick, Draw! dataset (Ref. [33]), Google LLC, used under the Creative Commons Attribution 4.0 International (CC BY 4.0) license with attribution. The samples were preprocessed (e.g., binarization/cropping/resizing) for this work. Example characters shown in this figure are from the EMNIST dataset (Ref. [34]), distributed via NIST and derived from NIST Special Database 19. The samples were used for research and were preprocessed (e.g., binarization/cropping/resizing) for this work.

To demonstrate the scalability of our morphological diffractive operators, we first focused on processing images with 320 × 320 pixels. Figure 4a shows the architecture of the morphological diffractive operators for processing large images up to 320 × 320 pixels. Importantly, the size of each diffractive layer was expanded to 640 × 640 neurons to effectively handle such image size. To achieve optimal image processing performance, we also conducted a parameter sweep of interlayer spacing (Supporting Information S1: Figure S13), which revealed that optimal performance was achieved with an interlayer spacing of 5λ. For training the scaled‐up diffractive network, we customized a hybrid dataset consisting of 50,000 images constructed by stitching 16 images of 80 × 80 pixels randomly selected from the EMNIST, gratings, and QuickDraw datasets into a 4 × 4 array. The test dataset was constructed in a similar manner but using images/styles that were unseen during training to reflect the generalizability of the model. Figure 4b shows some representative output of our scaled‐up diffractive erosion operator when blindly tested using stitched EMNIST and grating images. All processed images showed clear reduction in image features, as expected from the erosion process. For the EMNIST dataset, the SSIM between the processed output and the target image reached 0.713, whereas for the grating dataset, the SSIM was slightly lower at 0.643, likely due to the higher‐frequency step‐like features in the grating dataset, which were more challenging for optical approximation. We also tested the external generalizability of the model using the leaf images from QuickDraw, which were never used during training, with results shown in Supporting Information S1: Figure S13.

We further demonstrated the diffractive morphological processing of scaled‐up image inputs with 640 × 640 pixels. As with the 320 × 320 images, we conducted a parameter sweep for interlayer spacing (Supporting Information S1: Figure S14). The results show that for the 640 × 640 input size, optimal performance was achieved with an interlayer spacing of 5λ. The test dataset for the 640 × 640 task was constructed similarly to the 320 × 320 task but with 64 randomly selected 80 × 80 images from unseen categories, which were stitched into an 8 × 8 array. This larger dataset allowed us to evaluate the network's performance on even layer input images, providing further insight into its scalability and robustness. Figure 4b presents representative results from this task. For the EMNIST dataset, the average SSIM was 0.656, whereas for the gratings dataset, it was 0.483, which represents a slight decrease compared to the 320 × 320 task. This reduction in performance is attributed to the increased number of trainable units in the diffractive layers as the image size increases, leading to greater difficulty in network training [58]. These results demonstrate that the diffractive network is still capable of performing morphological transformations on such large‐scale inputs, highlighting its strong scalability.

### 2.6 Experimental Demonstration of All‐Optical Morphological Diffractive Operators

**FIGURE 5:**
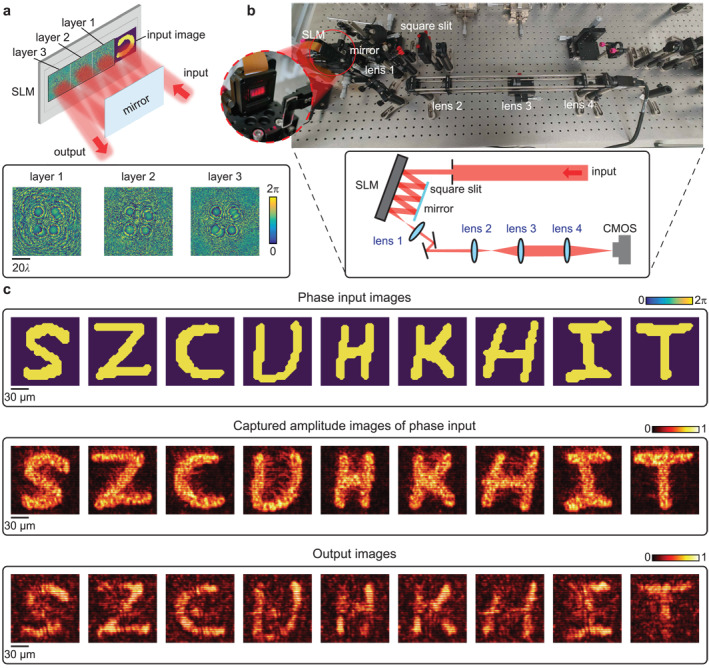
Experimental demonstration of diffractive morphological processing. (a) Scheme of the cascaded 3‐layer reflective diffractive network using mirror reflection and the optimized designs of the phase modulation layers. (b) A photo and a scheme depicting the optical setup for testing the morphological diffractive operators at a visible wavelength *λ* = 635 nm. The collimated laser beam was shaped by a square slit and directed to the SLM; after multiple reflections between the mirror and diffractive layers, the output was relayed and imaged onto a CMOS camera. (c) Representative experimental results: input phase images, amplitude‐mapped reference images, and experimentally captured outputs demonstrating concurrent phase imaging and morphological processing. Copyright: Example characters shown in this figure are from the EMNIST dataset (Ref. [34]), distributed via NIST and derived from NIST Special Database 19. The samples were used for research and were preprocessed (e.g., binarization/cropping/resizing) for this work.

We experimentally validated the designs of our morphological diffractive operators, which were realized in a multireflection mode using an SLM and a parallel mirror. Although the numerical designs were previously developed in a transmission‐mode form, the reflection‐mode implementation, which provided a compact proof‐of‐concept platform, could be trained to achieve equivalent performance. Figure 5 shows a scheme and the corresponding photo of our optical setup, as well as the optimized designs’ diffractive layers. A supercontinuum laser source (SC‐5, NKT Photonics) was spectrally filtered at a central wavelength of 635 nm, and a 4f filtering–expanding system was employed to spatially clean and collimate the beam. The resulting uniform illumination served as the light source of the diffractive network. A phase‐only SLM (RSL, China) carried the trained diffractive phase masks (see Methods section), and a 10‐mm mirror was inserted to fold the beam path, enabling multiple reflections between diffractive planes. Limited by the phase‐only modulation capabilities, the input images to be transformed were also represented by phase maps on the SLM region before the first diffractive layer. A square slit with a continuously adjustable aperture was positioned in front of the SLM to flexibly control the incident beam size. The beam was compressed to a 2.52 mm × 2.52 mm square to match the 700 × 700 active phase‐tuning units of the SLM diffractive layer. Each 4 × 4 pixel block of the SLM was binned as a single effective neuron, which relaxed alignment tolerance and reduced experimental errors caused by misregistration. The mirror was placed 40 mm away from the SLM surface, and the spacing between adjacent diffractive layers corresponded to 900 SLM pixels, ensuring sufficient diffraction between layers. Interlayer propagation was modeled using the off‐axis angular‐spectrum method (details in the Methods section).

The experimental implementation of the diffractive network is illustrated in Figure 5a; the incident collimated beam, after being clipped by the square slit, entered the first phase layer at an incident angle of 2.319°, acquired the encoded phase modulation, and was sequentially reflected to the following diffractive layers. After the third layer, the emerging field formed an output image representing the morphological transformation of the input phase pattern. The overall optical path and imaging chain are depicted in Figure 5b: A first 4f system relays the SLM output, whereas a second 4f system magnifies and projects the image onto the CMOS sensor. Before the experiment, the trained model was validated on the mixed EMNIST, QuickDraw, and custom gratings test sets to assess generalization. The simulated SSIMs reached 0.807 for EMNIST, 0.758 on average for QuickDraw categories, and 0.697 for gratings. Representative samples from these datasets were then selected for optical testing. Figure 5c shows part of the experimental results. For phase‐type inputs, direct quantitative comparison of phase and output fields is difficult; therefore, we used a pretrained single‐layer holographic converter to represent the input phase distribution in amplitude form, facilitating visual comparison. The experimental outputs clearly demonstrate that the diffractive network performed effective erosion of the EMNIST images, where the processed images showed reduced width in the strokes of the letters.

We also showed that the diffractive erosion operator could be trained to transform amplitude‐type images (Supporting Information S1: Figure S15). However, the experimental results exhibited a relatively low signal‐to‐noise ratio, which can be primarily attributed to imperfections in optical path alignment and residual aberrations of the reflective setup. Although additional random noise was intentionally introduced during the training stage to enhance model robustness, the diffractive network, being inherently linear, remains sensitive to angular misalignment and positional deviation between the spatial light modulator and the mirror. These imperfections collectively led to speckle‐like background noise and reduced image contrast in the measured outputs. Despite these imperfections, the diffractive network successfully accomplished its target tasks of phase imaging and morphological erosion. Consistent erosion was also achieved for amplitude‐encoded inputs, demonstrating reliable performance in practical optical environments.

## 3 Conclusions

We realized an all‐optical diffractive network framework for performing rapid, massively parallel morphological transformations of images. This framework leverages multilayer diffractive surfaces to manipulate the phase of the input optical fields, enabling fast and computer‐free visual information processing. Our results demonstrate that the diffractive network can effectively approximate basic morphological operations such as image erosion and dilation, with the flexibility to scale the size of the structuring elements for different transformation extents. Importantly, by adjusting the size of the morphological kernels, the system can perform various isotropic and anisotropic transformations, showcasing the wide applicability of this technology in different image processing tasks. We also explored the cascaded architecture of diffractive modules, which allows for the implementation of more complex morphological operations, such as opening and closing. Furthermore, our study highlights the high scalability of this system, not only for small‐sized images but also for large high‐resolution images, such as images with 320 × 320 and 640 × 640 pixels. Through extensive simulation and experimental validation, we demonstrated that our diffractive networks maintain strong performance across these large‐scale inputs, offering significant advantages over conventional algorithms.

The feasibility of the system was experimentally verified under visible‐light operation using a spatial light modulator (SLM)‐based multireflection setup. The morphological diffractive operators were realized by loading the optimized phase masks onto the SLM, enabling dynamic reconfiguration and accurate validation of the trained models without physical fabrication. This programmable platform eliminates the need for permanent diffractive elements, allowing rapid prototyping of diverse optical functions and task‐specific transformations. This SLM‐based all‐optical image processing framework may be further extended toward physically integrated implementations along two feasible device routes. First, microscale 3D diffractive elements can be fabricated using two‐photon polymerization printing [59], as demonstrated in recent studies on free‐space diffractive optical networks and compact multilayer optical components. Such resin‐based printed diffractive structures can be heterogeneously integrated with CMOS detector planes to form presensor optical computing modules [60], where morphological transformations are embedded directly in the optical front end prior to electronic readout. This approach is particularly attractive for low‐power edge perception systems and task‐specific visual preprocessing. In addition, metasurface‐based implementations using high‐index dielectric materials such as TiO_2_ or Si_3_N_4_ [61] can provide another promising route toward fully planar and lithographically scalable diffractive networks with submillimeter‐scale footprints. Besides, on‐chip diffractive networks integrated directly on planar photonic platforms [62] offer an alternative direction in which diffraction, propagation, and transformation are all implemented on chip, eliminating the need for macroscopic relay optics. Future work will therefore focus on translating the proposed morphological diffractive modules toward CMOS‐compatible presensor computing architectures and chip‐scale diffractive processors.

## 4 Methods

### 4.1 Forward Model of the All‐Optical Morphological Diffractive Operators

The all‐optical morphological transformation processor consisted of a series of diffractive layers (L=1,2,...,l) designed by deep learning. For such physical processes, free‐space propagation determined the light transport between (i) the input plane (L=0) and the diffractive surface (L=1), (ii) the diffractive surface (L=l−1) and the diffractive surface (L=l), and (iii) the diffractive surface (L=l) and the output plane (L=l+1). The free‐space propagation of the complex optical field profile in air (n=1) was modeled using the angular spectrum method based on the Rayleigh–Sommerfeld diffraction integral:

(1) $$u\left(x,y,z+d\right)=F^{-1}\left\{F\left\{u\left(x,y,z\right)\right\}H\left(f_{x},f_{y};d\right)\right\}$$

where u(x,y,z) is the input optical field, and u(x,y,z+d) is the optical field after propagation over an axial distance d. Operators F and $F^{-1}$ represent the 2D Fourier transform and inverse Fourier transform, respectively. $H\left(f_{x},f_{y};d\right)$ is the transfer function of free space, where $f_{x}$ and $f_{y}$ represent the spatial frequencies along the *x* and *y* directions, respectively. The transfer function was calculated by the following equation:

(2) $$H\left(f_{x},f_{y};d\right)=\left\{\begin{array}{ll} \exp\left\{jkd\sqrt{1-{\left(\frac{2\pi f_{x}}{k}\right)}^{2}-{\left(\frac{2\pi f_{y}}{k}\right)}^{2}}\right\}, & {f_{x}}^{2}+{f_{y}}^{2}\leq \frac{1^{2}}{\lambda^{2}}\\ 0, & {f_{x}}^{2}+{f_{y}}^{2}>\frac{1^{2}}{\lambda^{2}} \end{array}\right.$$

where *λ* is the illumination wavelength, $j=\sqrt{-1}$, and $k=\frac{2\pi }{\lambda }$ is the wavevector.

We set each diffractive layer to modulate only the phase of the transmitted optical field. The complex transmittance coefficient of each diffractive layer ($t^{l}\left(x,y\right)$) was written as follows:

(3) $$t^{l}\left(x,y\right)=\exp\left(j\phi^{l}\left(x,y\right)\right)$$

where $\phi^{l}\left(x,y\right)$ represent the modulation of the optical field phase by the neuron at (x,y) on the diffractive layer.

By a combination of Equations (1) and (3), the output complex optical field distribution after passing through any number of diffractive layers could be calculated.

### 4.2 Training Loss Functions

The phase modulation coefficients of each diffractive layer were obtained in a deep learning‐based training process by (1) calculating the loss function based on the comparison between the output light field and the ground‐truth optical field and (2) adjusting the network design through backpropagation of the losses. In the training of the diffractive network for erosion transformations, we used the normalized mean squared error (NMSE) between the output optical field (O(x,y)) and the ground‐truth intensity pattern (G(x,y)) as the loss function, which was defined as follows:

(4) $$\text{NMSE}\left(O\left(x,y\right),G\left(x,y\right)\right)=\frac{1}{P_{\text{tot}}}\sum_{x,y}{\left(\frac{O\left(x,y\right)}{\max\left(O\left(x,y\right)\right)}-G\left(x,y\right)\right)}^{2}$$

where $P_{\text{tot}}$ represent the total number of pixels in each image. The normalization term max(O(x,y)) ensured that the output intensity O(x,y) was scaled to the same dynamic range as the target pattern, preventing global intensity variations or optical power fluctuations from dominating the loss calculation.

In the all‐optical image dilation diffractive networks, network performance obtained using only NMSE as the loss function was undesirable. To ensure the fidelity and uniformity of the image edge after enlargement, we used a hybrid loss function of NMSE and the Pearson correlation coefficient (PCC), where PCC was defined as follows:

(5) $$\text{PCC}\left(O\left(x,y\right),G\left(x,y\right)\right)=\frac{\sum_{x,y}\left(O\left(x,y\right)-\bar{O}\right)\left(G\left(x,y\right)-\bar{G}\right)}{\sqrt{\sum_{x,y}{\left(O\left(x,y\right)-\bar{O}\right)}^{2}{\left(G\left(x,y\right)-\bar{G}\right)}^{2}}}$$

where $\bar{O},\bar{G}$ represent the mean values of the output optical field and ground‐truth optical field, respectively. The hybrid loss function is defined as follows:

(6) $$\text{Loss}\left(O,G\right)=\text{NMSE}\times w_{\text{NMSE}}+\left(1-\text{PCC}\right)\times w_{\text{PCC}}$$

where *w*_NMSE_and *w*_PCC_ represent the different weights assigned to NMSE and PCC.

### 4.3 Preparation of Image Datasets

For both erosion and dilation networks demonstrated in Figures 2 and 3, we trained the morphological diffractive operators using a processed hybrid dataset consisting of three parts: (i) 37,600 handwritten digits and letters randomly sampled from one third of the EMNIST training set, (ii) 30,000 sketch‐style images from 6 object categories (bee, door, donut, grape, ladder, and T‐shirt) in the QuickDraw dataset with 5000 samples per category, and (iii) 5000 customized line‐grating patterns with randomized stripe widths (8–14 pixels) and orientations (0–180°). This hybrid dataset was designed to enable accurate morphological quantification and promote the generalization of the model when processing diverse visual objects. To standardize input resolution, all images were upsampled to 80 × 80 pixels using nearest‐neighbor interpolation and normalized prior to further processing. For evaluating the generalization capability of the diffractive network, we constructed a separate test dataset consisting of previously unseen samples: (i) 6267 images randomly sampled from one third of the EMNIST test set, (ii) 25,000 QuickDraw images from 5 new object categories (circle, cloud, lightning, leaf, and eyeglasses) that were not included in the training set, and (iii) 5000 grating patterns generated with stripe widths and rotation angles not present in the training set. All test images underwent the same upsampling and normalization procedures to ensure consistency.

For isotropic morphological operations, a 5 × 5 all‐ones matrix was used as the structuring element. All images in the training and test datasets were first upsampled to 80 × 80 pixels and then normalized. To obtain a smooth initial input field for optical fitting, each image was preprocessed with a single dilation operation using the 5 × 5 structuring element. The resulting image served as the input optical field. Subsequently, the morphological operation (erosion or dilation) was applied again using the same 5 × 5 structuring element to generate task‐specific target outputs for the isotropic diffractive network.

For fitting different structuring element transformations, the same upsampling and preprocessing procedure was applied. For variable‐sized isotropic operations, the input field generated from a preliminary 5 × 5 dilation was then subjected to erosion or dilation using larger kernels (e.g., 7 × 7 or 9 × 9) to construct target outputs representing varying degrees of morphological change. For anisotropic operations, the preprocessed input field was transformed using non‐square structuring elements, including 1 × 5 and 5 × 1 matrices, to selectively induce morphological changes in vertical or horizontal directions, respectively. The resulting images served as the target outputs for training anisotropic diffractive networks.

For high‐resolution tasks, we generated large images by stitching smaller preprocessed patches. For 320 × 320 images, 16 randomly sampled 80 × 80 input images were arranged in a 4 × 4 array. For 640 × 640 images, 64 images were arranged in an 8 × 8 array. For the test datasets, unseen images from the same categories were randomly selected and similarly stitched to construct high‐resolution evaluation inputs, ensuring that the network's performance on large‐scale images could be assessed independently of training exposure.

For noise‐robust evaluation, we first generated input images using the preprocessing methods described above. Salt noise was then added at varying SNRs. Unlike conventional unit‐sized salt noise, the added noise was generated with a variable size ranging from 1 × 1 to 3 × 3 pixels to better simulate realistic noise patterns. These noisy images were used to test the diffractive networks' performance in denoising and morphological gradient extraction tasks.

Unless otherwise stated in the figure legends, all schematics, system diagrams, and result visualizations were created by the authors for this work and are 2026 The Authors. Example input images used for demonstrating the diffractive morphological operators were drawn from publicly available datasets: EMNIST (Ref. [34], distributed via NIST and derived from NIST Special Database 19) and Quick, Draw! (Ref. [33], Google LLC, used under the Creative Commons Attribution 4.0 International (CC BY 4.0) license). Any dataset samples shown in the figures were used with attribution and were preprocessed (e.g., binarization/cropping/resizing) solely for scientific visualization and training. No endorsement by the data providers is implied.

### 4.4 Blockwise Noise Model and Evaluation Metrics

To further evaluate the denoising capability of our diffractive system under structured disturbances, a blockwise impulse noise model was introduced. Unlike conventional pixel‐level salt‐and‐pepper noise, this model generates compact bright patches to simulate local overexposure artifacts commonly observed in optical imaging. For an input image I(x,y)ϵ[0,1], the number and intensity of noise patches are governed by a signal‐to‐noise ratio parameter $\text{SN}R_{\text{Bnoise}}$ (in dB). The average signal power is estimated as the mean image intensity:

(7) $$P_{S}=\frac{1}{N}\sum_{x,y}I\left(x,y\right)$$

and the corresponding noise power is computed as follows:

(8) $$P_{N}=\frac{P_{S}}{10^{\left(\text{SN}R_{\text{Bnoise}}/10\right)}}$$

Given the total number of pixels N, the expected number of noise blocks is given as follows:

(9) $$N_{b}=P_{n}\times N$$

Each block is randomly sampled with a size:

(10) $$b\epsilon \left[1,b_{\max}\right]$$

and a random top‐left coordinate $\left(x_{0},y_{0}\right)$.

All pixels within the square region

(11) $$\left\{\begin{array}{c} x_{0}\leq x\leq x_{0}+b\\ y_{0}\leq y\leq y_{0}+b \end{array}\right.$$

are set to 1, representing bright noise clusters.

In our experiments, $b_{\max}=3$ was used for both training and testing datasets. This design introduces locally clustered degradation, offering a more challenging denoising scenario than uncorrelated random noise.

To quantitatively assess the denoising performance, the peak signal‐to‐noise ratio metric was employed [63]. The PSNR evaluates the reconstruction fidelity between a clean target image $y_{i}$ and its denoised counterpart $\hat{y_{i}}$. It is defined as follows:

(12) $$\text{PSNR}=10\;\log_{10}\left(\frac{1}{\frac{1}{N}{\sum_{i=1}^{N}\left|y_{i}-\frac{\sum_{i=1}^{N}y_{i}\hat{y_{i}}}{\sum_{i=1}^{N}{\hat{y_{i}}}^{2}}\hat{y_{i}}\right|}^{2}}\right)$$

where N denotes the total number of pixels.

A higher PSNR indicates better denoising quality and closer similarity to the ground‐truth image.

### 4.5 Training and Digital Implementation

In the simulation, the minimum feature size of each diffractive layer was set to 0.47λ, and the physical size of a single pixel in the input and output images was 0.94λ. For both isotropic and anisotropic morphological transformations, each layer contained 160 × 160 modulation units, and the network consisted of 5 diffractive layers. To improve simulation accuracy, the computational grid resolution was set to 0.235λ so that each minimum modulation unit was represented by four grid points. Additionally, zero padding was applied to expand the computational plane from 320 × 320 to 620 × 620 grid points, further enhancing the accuracy of optical propagation modeling. For cascaded tasks, multiple morphological diffractive operators—performing either the same or different operations—were connected sequentially, such that the complex‐valued output field of the preceding network served directly as the input for the next network.

To accommodate high‐resolution images, the size of the diffractive layers was increased to enhance the modulation capacity of the diffractive networks. For 320 × 320 pixel input images, while keeping the computational grid resolution, input/output pixel size, and minimum feature size unchanged, each layer was expanded to 640 × 640 modulation units, maintaining 5 layers. For 640 × 640 pixel images, each layer was further increased to 1280 × 1280 modulation units to achieve better fitting and transformation accuracy.

The model was optimized using the Adam optimizer with a learning rate of 0.001 and a batch size of 60. All simulations were conducted using Python 3.11 and PyTorch 2.5.0 on a workstation equipped with a single NVIDIA GeForce RTX 4090D GPU. A typical training session of 200 epochs required approximately 6 h.

### 4.6 Details of the Experiment

To better fit the experimental model, we introduced an additional diffraction‐efficiency loss term, $\text{Los}s_{\text{diff}}$:

(13) $$\text{Los}s_{\text{diff}}=\mu \left(1-\frac{\sum O_{\text{img}}}{T_{\text{eff}}\sum I_{\text{tol}}}\right)$$

where μ denotes a weighting constant, $I_{\text{tol}}$ represents the total optical intensity of the input field, $O_{\text{img}}$ is the integrated intensity within the target imaging region of the output field, and $T_{\text{eff}}$ is the threshold efficiency. In this experiment, $T_{\text{eff}}$ was empirically set to 20%, and $\text{Los}s_{\text{diff}}$ was activated only when the diffraction efficiency fell below this threshold.

Because the incident beam impinges on the SLM at an oblique angle, we employed an off‐axis angular spectrum method instead of the conventional angular spectrum propagation to accurately model the tilted‐beam free‐space diffraction [64], expressed as follows:

(14) $$G_{\text{off}}\left(f_{xs},f_{ys};z\right)=G\left(f_{xs}+f_{x0},f_{ys}+f_{y0};0\right)\times \exp\left\{j2\pi \left(f_{xs}x_{0}+f_{ys}y_{0}\right)+jkz\sqrt{1-{\left(\frac{2\pi \left(f_{x0}+f_{xs}\right)}{k}\right)}^{2}-{\left(\frac{2\pi \left(f_{y0}+f_{ys}\right)}{k}\right)}^{2}}\right\}$$

Here, $G_{\text{off}}\left(f_{xs},f_{ys};z\right)$ is an off‐axis spatial frequency spectrum. $f_{xs}$ and $f_{ys}$ correspond to the displaced spatial frequencies along the *x* and *y* directions, respectively, whereas $f_{x0}$ and $f_{y0}$ represent the carrier frequencies in the spatial domain.

In simulation, we used input images from the same 80 × 80 dataset as in the previous training tasks, expecting output images of the same size. However, preliminary experiments revealed degraded image quality due to optical imperfections. To improve system robustness, we augmented the input images with salt noise and found that setting the input SNR to approximately 14 dB achieved the best trade‐off—enhancing model robustness and output fidelity without significant degradation from excessive input noise. The minimum SLM pixel pitch is 3.6 μm, which we adopted as the simulation's smallest sampling grid. Each diffractive layer consisted of 160 × 160 neurons, and because each neuron physically corresponded to a 4 × 4 block of SLM pixels, its physical size was set to 14.4 μm. The same pixel size (14.4 μm) was used for both the input and output planes. To ensure geometric alignment, the input and output images were zero‐padded to match the layer dimension, and an additional 100‐pixel margin was simulated around each diffractive layer to maintain consistency between simulation and experiment. During the experiment, two motorized rotation stages were used to finely control the angular alignment of the SLM and the mirror. Additionally, both components were mounted on independent three‐axis translation stages, ensuring that the SLM and the mirror were precisely positioned at the same height and separated by the designed distance. This alignment guaranteed that, during multiple reflections, the optical beam precisely covered the corresponding diffractive layers on the SLM surface. These measures collectively ensured close agreement between simulated and experimental conditions, enabling reliable validation of the diffractive network's optical performance.

## Author Contributions

J.H. conceived the initial idea. Y.S. performed the design and training of the model assisted by J.H., G.Q., Y.W., F.W. and S.W. and performed the analysis of the resulting models. Y.S., J.H. and G.Q. conducted the analysis of the resulting models. Z.Z., Q.R. and H.W. contributed to the device preparation. Y.S. and F.W. conducted the imaging test. All the authors contributed to the preparation of the manuscript. J.H., H.M, and J.G. supervised the research.

## Funding

This research was supported by the National Natural Science Foundation of China (Grant Nos. 62535008, 62405076, and 12404442); the Guangdong Basic and Applied Basic Research Foundation (Nos. 2023A1515110685 and 2025A1515011713); the Guangdong Provincial Key Laboratory of Semiconductor Optoelectronic Materials and Intelligent Photonic Systems (No. 2023B1212010003); the Guangdong Provincial Quantum Science Strategic Initiative (Nos. GDZX2306002 and GDZX2506001); the Shenzhen Science and Technology Program (Nos. JCYJ20240813113603005, JCYJ20240813104929039, JCYJ20250604145557075, and JCYJ20250604141202003). J.H. was also supported by the Guangdong Major Project of Basic Research (No. 2025B0303000008) and the research funding of the Key Laboratory of Photonic Technology for Integrated Sensing and Communication, Ministry of Education, Guangdong University of Technology, Guangzhou. J.G. and C.H. also acknowledge support from the 1+1+1 CUHK‐CUHK(SZ)‐GDSTC Joint Collaboration Fund (No. 2025A0505000071). J.G. acknowledges support from the 1+1+1 CUHK‐CUHK(SZ)‐GDSTC Joint Collaboration Fund (No. 2025A0505000052).

## Conflicts of Interest

The authors declare no conflicts of interest.

## Supporting information


Supporting Information S1

## Data Availability

The data that support the findings of this study are available from the corresponding author upon reasonable request.
