# Correction to “All‐Optical Diffractive Operators for Rapid, Computer‐Free Morphological Transformations”

**DOI:** 10.1002/nap2.70073

**Published:** 2026-03-26

Sun, Yuxiang, Fenglei Wang, Jing Han, et al. “All‐Optical Diffractive Operators for Rapid, Computer‐Free Morphological Transformations,” *Nanophotonics* e70031 (2026). https://doi.org/10.1002/nap2.70031.

In Figure 5, the scale bar label of the published article was incorrect. The label should have read: “300 μm.” In addition, the corrected Figure S15 is provided in the latest version of the Supporting Information. It should be emphasized that this mistake does not influence the major claims and novelty of the work. The revised corrected Figure 5 is presented below.

We apologize for this error.


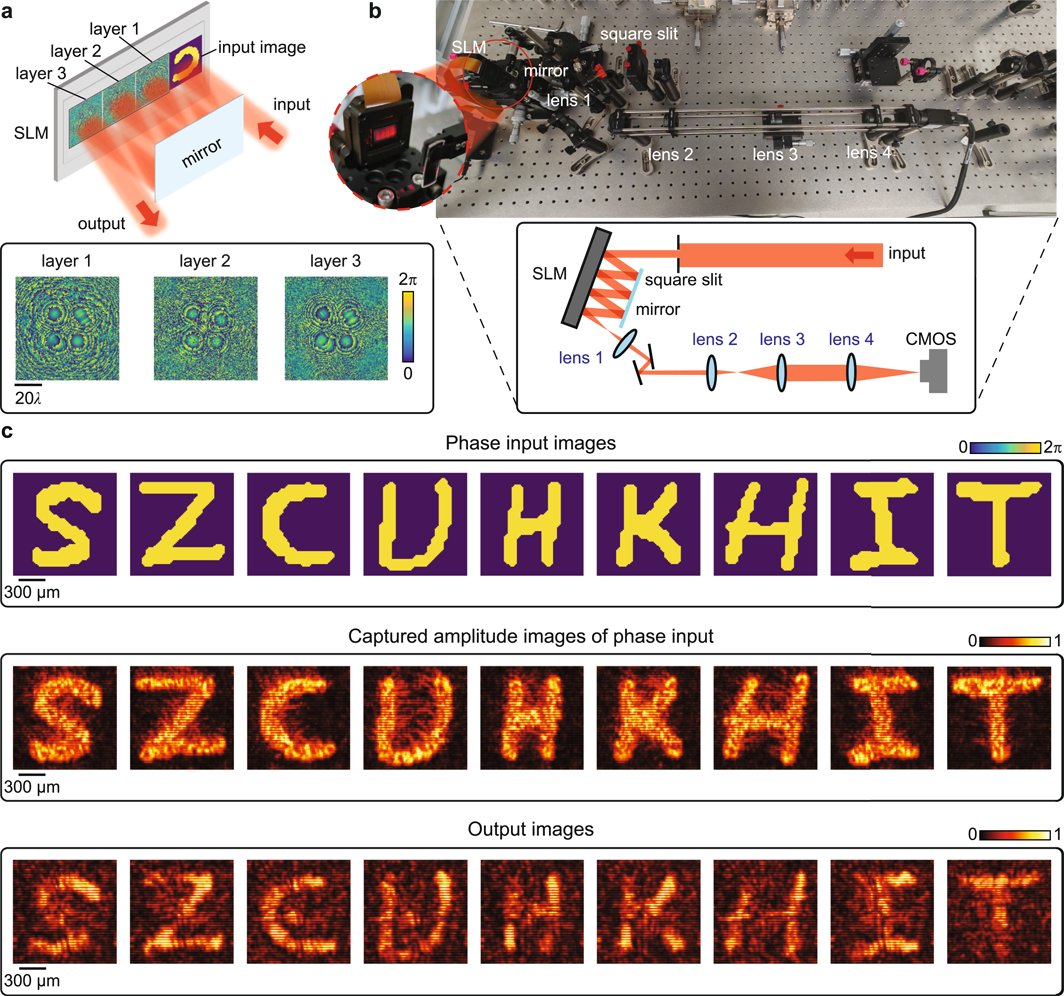


## Supporting information


Supporting Information S1

