# Supplementary material for: All‐Optical Diffractive Operators for Rapid, Computer‐Free Morphological Transformations
Source: Nanophotonics. 2026 Feb 22;15(4):e70031. doi: 10.1002/nap2.70031 (PMC12965033; doi:10.1002/nap2.70031)
Supplement: Supplementary file 1 — Supporting Information S1 [file NAP2-15-e70031-s001.pdf]

SUPPORTING INFORMATION

All-Optical Diffractive Operators for Rapid, Computer-Free Morphological Transformations

Yuxiang Sun,[1,2] *Fenglei Wang,*[1] *Jing Han,*[1] *Geyang Qu,*[1] *Zezheng Zhang,*[1] *Yan Wei,*[3] *Chuang Yang,*[1] *Qifeng Ruan,*[1] *Shengjie Wang,*[4] *Heming Wei,**[3] *Chaoran Huang,*[5] *Jun Guan,**[2] *Jingtian Hu,**[1,6,7]

[1]Ministry of Industry and Information Technology Key Lab of Micro-Nano Optoelectronic Information System, Guangdong Provincial Key Laboratory of Semiconductor Optoelectronic Materials and Intelligent Photonic Systems, Harbin Institute of Technology, Shenzhen 518055, P. R. China

[2]School of Science and Engineering, The Chinese University of Hong Kong (Shenzhen), Shenzhen, Guangdong, 518172, P. R. China

[3]Shanghai University, Shanghai 200444, P. R. China

[4]New York University Shanghai, Shanghai 200124, P. R. China

[5]Department of Electronic Engineering, The Chinese University of Hong Kong, Shatin, Hong Kong SAR, P. R. China.

[6]Quantum Science Center of Guangdong-Hong Kong-Macao Greater Bay Area, Shenzhen 518055, P. R. China

[7]Key Laboratory of Photonic Technology for Integrated Sensing and Communication, Ministry of Education, Guangdong University of Technology, Guangzhou 510006, China.

*e-mail: hujingtian@hit.edu.cn

Table of Content for Supplementary Materials

Effect of inter-layer spacing on diffractive operators performance S3

Performance of diffractive erosion operators with varying layer configurations S6

Performance analysis of diffractive operators with varying lateral dimensions S8

Quantitative evaluation of the diffractive operators on isotropic erosion tasks S10

Quantitative evaluation of the diffractive operators on isotropic dilation tasks S11

Phase distribution maps of erosion and dilation operators for denoising tasks S13

External generalization to real surface-defect images... S15

Robustness analysis of cascaded diffractive networks under lateral misalignment........... S17

Controlled vs. free-space propagation in morphological erosion.. S18

Generalization of all-optical processors across morphological variations S22

Iterative and cascaded diffractive morphological transformations S23

All-optical morphological transformations of anisotropic structuring elements................ S24

Scalability and interlayer-spacing effects in diffractive operators (640×640)................... S27

Scalability and interlayer-spacing effects in diffractive operators (1280×1280)................. S28

Diffractive morphological erosion of amplitude-encoded images S30

Diffractive morphological processors for isotropic transformations

To optimize the performance of the diffractive networks for isotropic erosion and dilation tasks, we conducted a systematic parameter sweep of the inter-layer spacing between diffractive surfaces. The spacing was varied from 5λ to 100λ, including the distances from the input plane to the first layer and from the last layer to the detector (**Figures S1a,c**). For each spacing configuration, we evaluated the output quality using the structural similarity index measure (SSIM) between the predicted and target eroded/dilated images. The test datasets used to evaluate the generalization performance include: the EMNIST test set, a custom-built random gratings dataset

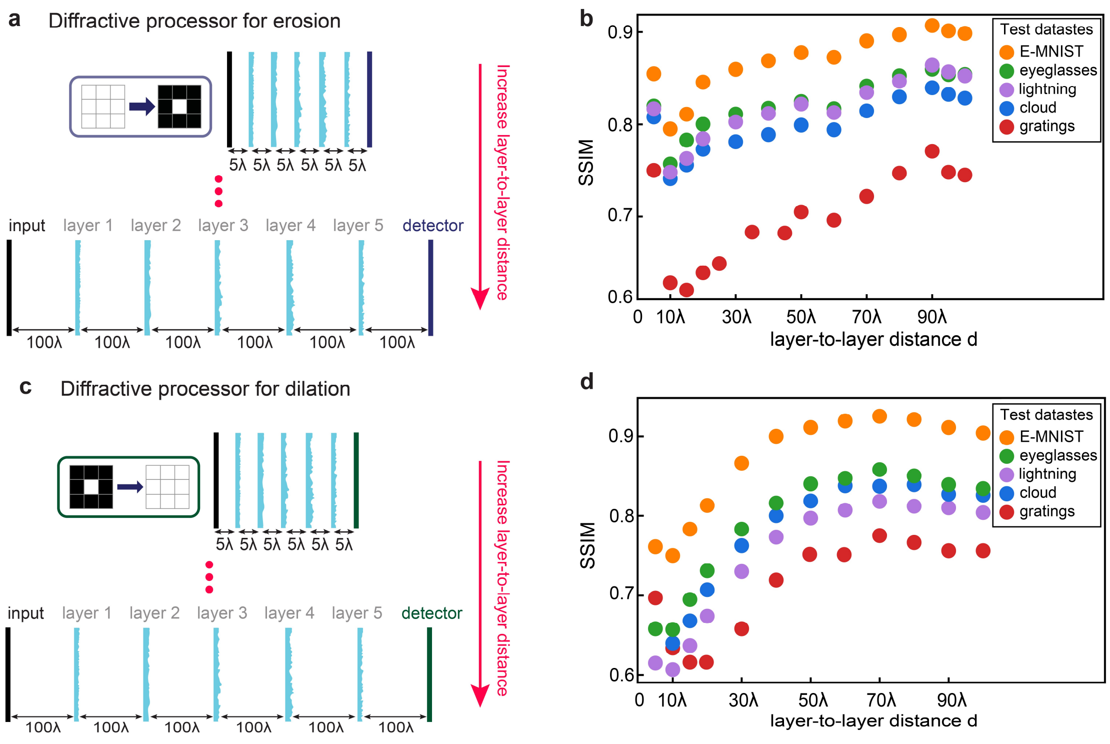

Figure S1: Impact of inter-layer spacing on imaging performance across different diffractive optical processors. (**a**) For image erosion tasks, the inter-layer spacing of diffractive layers is systematically varied from 5λ to 100λ. (**b**) Performance evaluation using unseen test datasets reveals that the SSIM reaches its peak at 90λ spacing, corresponding to optimal visual fidelity. (**c**) For image dilation tasks, diffractive layers maintain identical spacing configurations ranging from 5λ to 100λ. (**d**) Comprehensive testing on model-unseen datasets demonstrates maximum SSIM performance at 70λ spacing, indicating task-specific optimal configurations.

with unseen grating widths, and three unseen categories from the QuickDraw dataset — Eyeglasses, Lightning, and Cloud — none of which were used during training.

We observed that the EMNIST test set consistently yielded higher SSIM scores across spacing values, while the gratings dataset performed the worst. This discrepancy can be attributed to the fact that QuickDraw sketches exhibit more abstract shapes with sparse edge information, making them harder for the diffractive system to reconstruct accurately. The gratings images, composed of high-frequency, sharply defined patterns, pose additional challenges due to the network's limited ability to model abrupt optical transitions.

Quantitatively, for erosion tasks, the EMNIST dataset maintained SSIM values between 0.79 and 0.90, peaking at 0.897 at 95λ, while the gratings dataset reached only 0.748, yielding an average gap of approximately 0.15–0.17. The QuickDraw subclasses (Cloud, Eyeglasses, Lightning) exhibited moderate performance, with SSIM values ranging from 0.74 to 0.86 depending on propagation distance. The optimal SSIM for erosion tasks occurred at 90λ spacing (**Figure S1b**).In contrast, for dilation tasks, the overall SSIM values were slightly lower due to the more challenging edge-expansion operation. The best result again appeared in the EMNIST dataset, reaching a maximum SSIM of 0.821 at 80λ, whereas gratings peaked at 0.668, corresponding to a similar inter-dataset difference of $\approx$ 0.15. The QuickDraw subclasses showed smoother trends, with their SSIM values saturating around 0.70 ‒ 0.75 at optimal spacing distances (**Figure S1d**). These results confirm a clear task-dependent optimal configuration, with erosion networks favoring 90λ and dilation networks achieving their peak performance at 70λ, demonstrating consistent and interpretable morphological behavior across different datasets.

To investigate the performance-depth trade-off in diffractive processors, we conducted a systematic analysis by varying the number of diffractive layers from 2 to 7 while keeping the inter-

layer spacing and the physical dimensions of the diffractive elements fixed (**Figure S2a**). This experiment was designed to validate prior evidence suggesting that deeper free-space networks benefit from enhanced representation capacity, improved inference accuracy, and increased optical contrast. Quantitatively, the SSIM of the EMNIST dataset increased sharply from 0.572 (at 2 layers) to 0.893 (at 5 layers), representing an improvement of +0.321, before slightly dropping to 0.852 when the depth reached 7 layers. The gratings dataset exhibited a similar trend, rising from 0.234 to 0.769 (+0.535) and then falling to 0.667. Among the QuickDraw subclasses, QD-Cloud, QD-Eyeglasses, and QD-Lightning peaked at 0.862, 0.871, and 0.870 at 5 layers, respectively—an average SSIM gain of about 0.37–0.39 relative to the 2-layer baseline. Beyond five layers, performance consistently declined by $\approx 0.02 - 0.05$ across all datasets.

These results show that increasing the number of diffractive layers improves performance initially, with the SSIM peaking at five layers across most test datasets (**Figure S2b**). However, further increasing the depth beyond this point leads to performance degradation. This decline may be attributed to over-parameterization and slower convergence, where deeper networks struggle to reach optimal configurations within the same number of training epochs. Visual examples in **Figure S2c** highlight this effect, showing more accurate erosion outcomes with intermediate depth and diminishing returns—or even degradation—with excessive network depth.

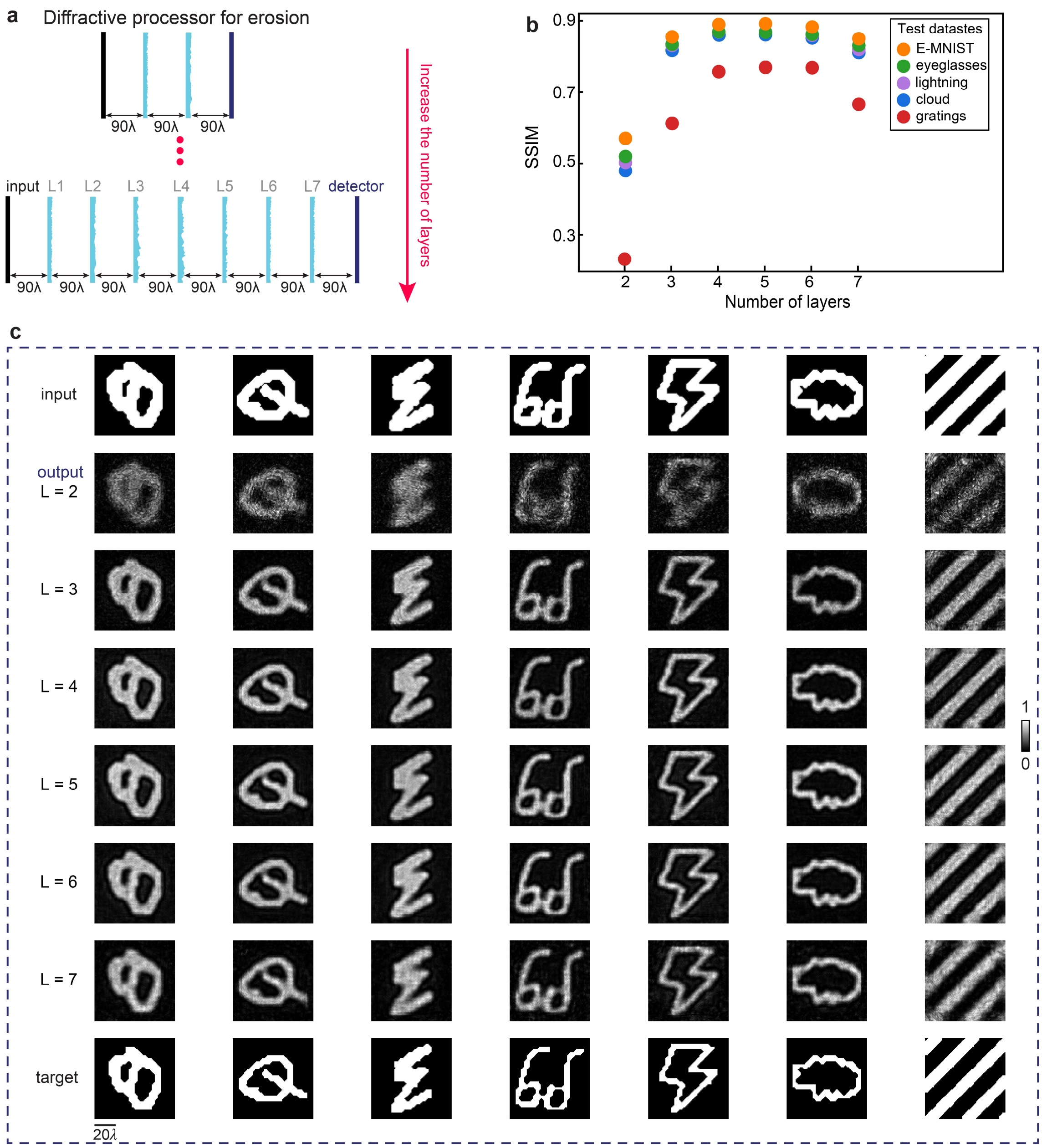

Figure S2: Performance analysis of image erosion diffractive processors with varying layer configurations. (**a**) Systematic investigation of layer count effects while maintaining constant inter-layer spacing and diffractive element dimensions. (**b**) Performance evaluation reveals an optimal architecture with 5 layers, where the SSIM initially increases with the layer count before declining due to over-parameterization effects. (**c**) Representative outputs demonstrate the processors' generalization capabilities across unseen test datasets, showcasing their ability to handle novel optical field information with different layer configurations.

To evaluate the influence of lateral resolution on the performance of diffractive processors, we systematically varied the number of neurons per layer (N × N) from 160 to 260 while maintaining a constant 5-layer depth and an inter-layer spacing of 90λ (**Figure S3a**). This experiment isolates the effect of lateral dimension scaling under fixed axial geometry and training conditions. Quantitatively, the SSIM values of the EMNIST dataset remained nearly constant, fluctuating only from 0.726 to 0.731 across all tested neuron counts, indicating that higher lateral sampling density brings negligible benefit once a sufficient spatial resolution is reached. The gratings dataset showed a similar saturation trend, varying slightly between 0.836 and 0.871, while the QuickDraw subclasses (QD-Cloud, QD-Eyeglasses, and QD-Lightning) maintained SSIM levels of 0.846–0.879, 0.871–0.881, and 0.648–0.655, respectively. As shown in **Figure S3b**, the overall improvement margin across the entire range (160 to 260 neurons) was less than 0.01 for most datasets, corresponding to an average relative gain below 1.5 %.

These results confirm that enlarging the diffractive layer's lateral size yields diminishing performance returns once the spatial sampling threshold is met. This observation suggests that optical morphological processors possess intrinsic resolution sufficiency, where excessive neuron counts mainly increase computational cost without tangible improvement in structural similarity. Representative reconstructions in **Figure S3c** further verify that the erosion quality remains visually consistent across all tested lateral dimensions.

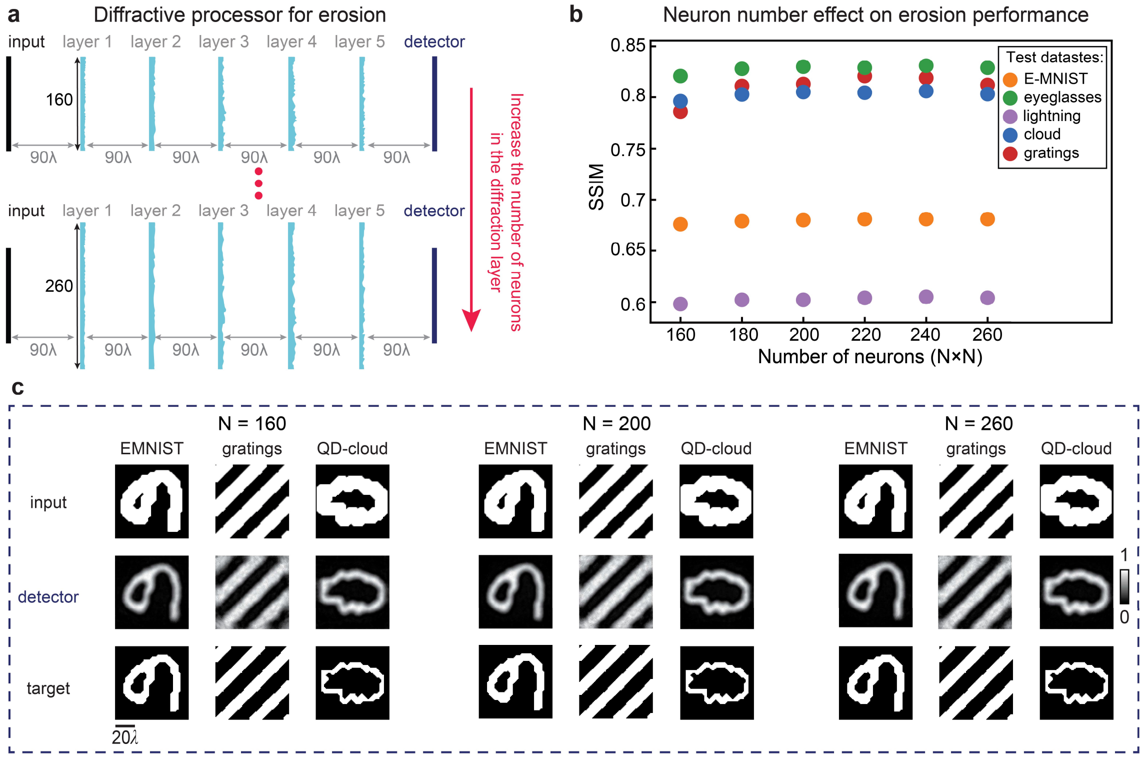

Figure S3: Performance analysis of diffractive processors with varying lateral dimensions. (**a**) Schematic comparison of diffractive processors with different neuron counts per layer (260 vs. 160), maintaining constant 5-layer depth and 90λ inter-layer spacing. (**b**) SSIM performance across neuron numbers (160-260) shows stable trends for all test datasets, with minimal improvement observed as lateral dimensions increase. (**c**) Representative erosion outputs for N= 160, 200, and 260 demonstrate consistent morphological processing quality across EMNIST, gratings, and QD-cloud datasets, indicating saturation of performance gains beyond moderate neuron counts.

To quantitatively evaluate the isotropic behavior of diffractive morphological processors, we conducted controlled experiments for both erosion and dilation tasks using a test set of 5,000 synthetically generated binary gratings with random linewidths and two orientations: 0°, 90° (**Figures S4–S5**). These gratings were unseen during training and were used to assess the spatial generalization of the learned morphological kernels.

For the erosion processor (**Figure S4a**), each input image was passed through a diffractive network trained to approximate an isotropic (5×5) erosion kernel. The output linewidths were measured to compute the reduction relative to the original inputs. As shown in **Figure S4b**, the

horizontal axis represents the linewidth difference (input – output linewidth, in pixels), and the vertical axis denotes the number of samples per linewidth bin. Gaussian-fitted histograms from 1,000 randomly selected samples reveal consistent average linewidth reductions of 3.65 ± 0.42 pixels for 0° gratings and 3.67 ± 0.46 pixels for 90° gratings, closely matching the target erosion values of 3.72 ± 0.40 and 3.73 ± 0.41 pixels, respectively. Representative input, output, and target comparisons (**Figure S4c**) confirm that the network performs uniform erosion across spatial directions, demonstrating isotropic transformation fidelity.

Similarly, the dilation processor (**Figure S5a**) was trained using the same architecture and evaluated on identical test conditions. **Figure S5b** presents the corresponding histograms of linewidth difference (output – input linewidth, in pixels) versus number of samples. The fitted Gaussian distributions yield mean linewidth expansions of 3.23 ± 0.47 pixels (0°) and 3.31 ± 0.44 pixels (90°), which agree well with the target dilation magnitudes of 3.65 ± 0.43 and 3.68 ± 0.42 pixels. Representative results in **Figure S5c** show consistent widening of bright regions regardless of pattern orientation.

Together, these results confirm that both diffractive erosion and dilation processors exhibit robust, orientation-independent behavior, achieving near-identical morphological transformations for horizontal and vertical gratings. This demonstrates that the learned diffractive kernels are effectively isotropic and capable of accurately modeling direction-invariant morphological operations in free-space optical networks.

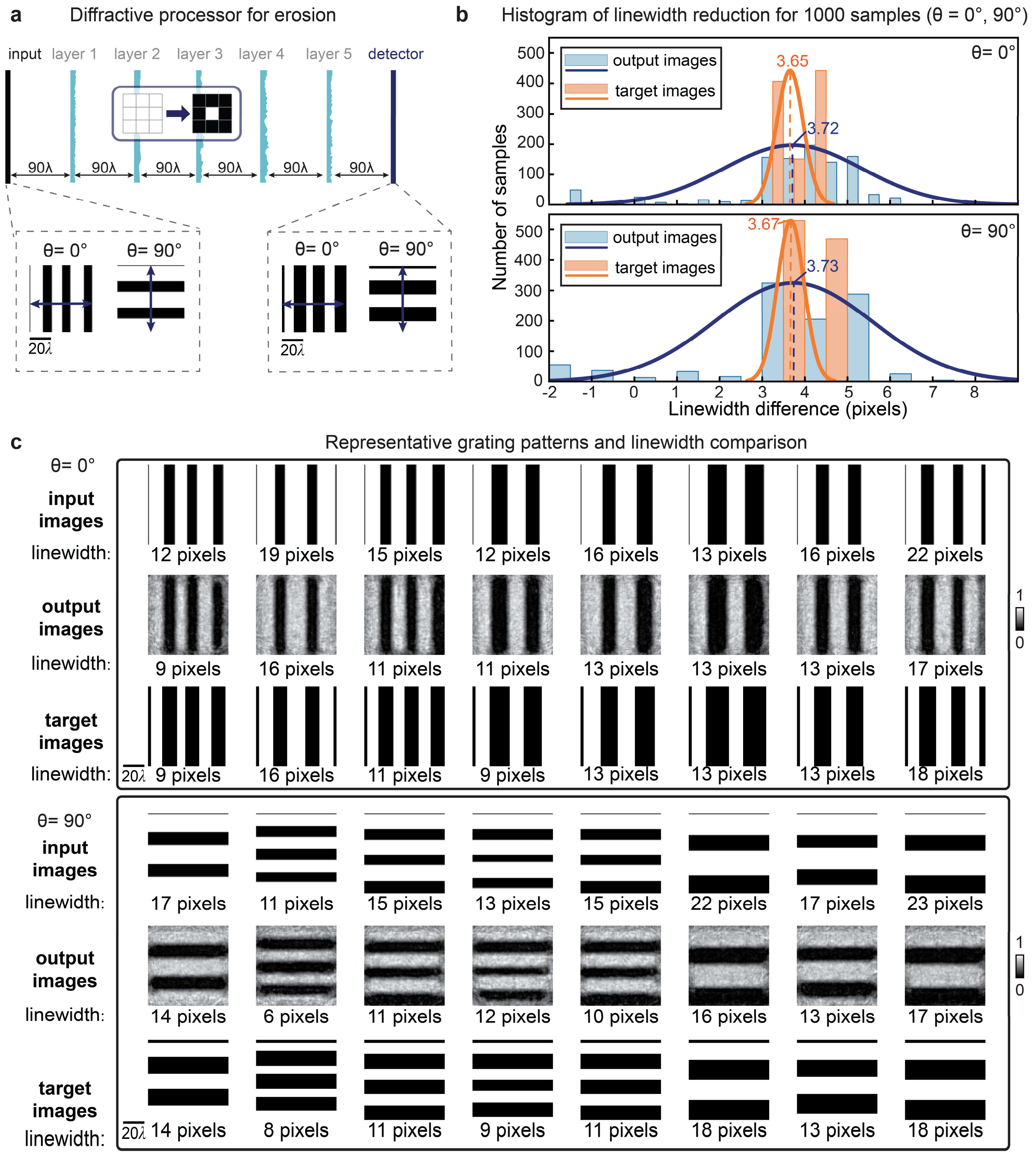

Figure S4: Quantitative evaluation of the diffractive processor on isotropic erosion tasks. (**a**) Schematic of the five-layer diffractive network trained to approximate a 5×5 isotropic erosion kernel. The input consists of gratings with varying orientations (0°, 45°, 90°, and 325°) to evaluate the directional generalization of the learned morphological operation. (**b**) Average linewidth reduction across different grating orientations after propagation through the same trained diffractive network. Dashed lines indicate the theoretical target values. (**c**) Representative input, output, and target patterns from the test set. Linewidth changes before and after erosion are shown for various grating directions, validating the processor's ability to approximate erosion behavior across spatial orientations.

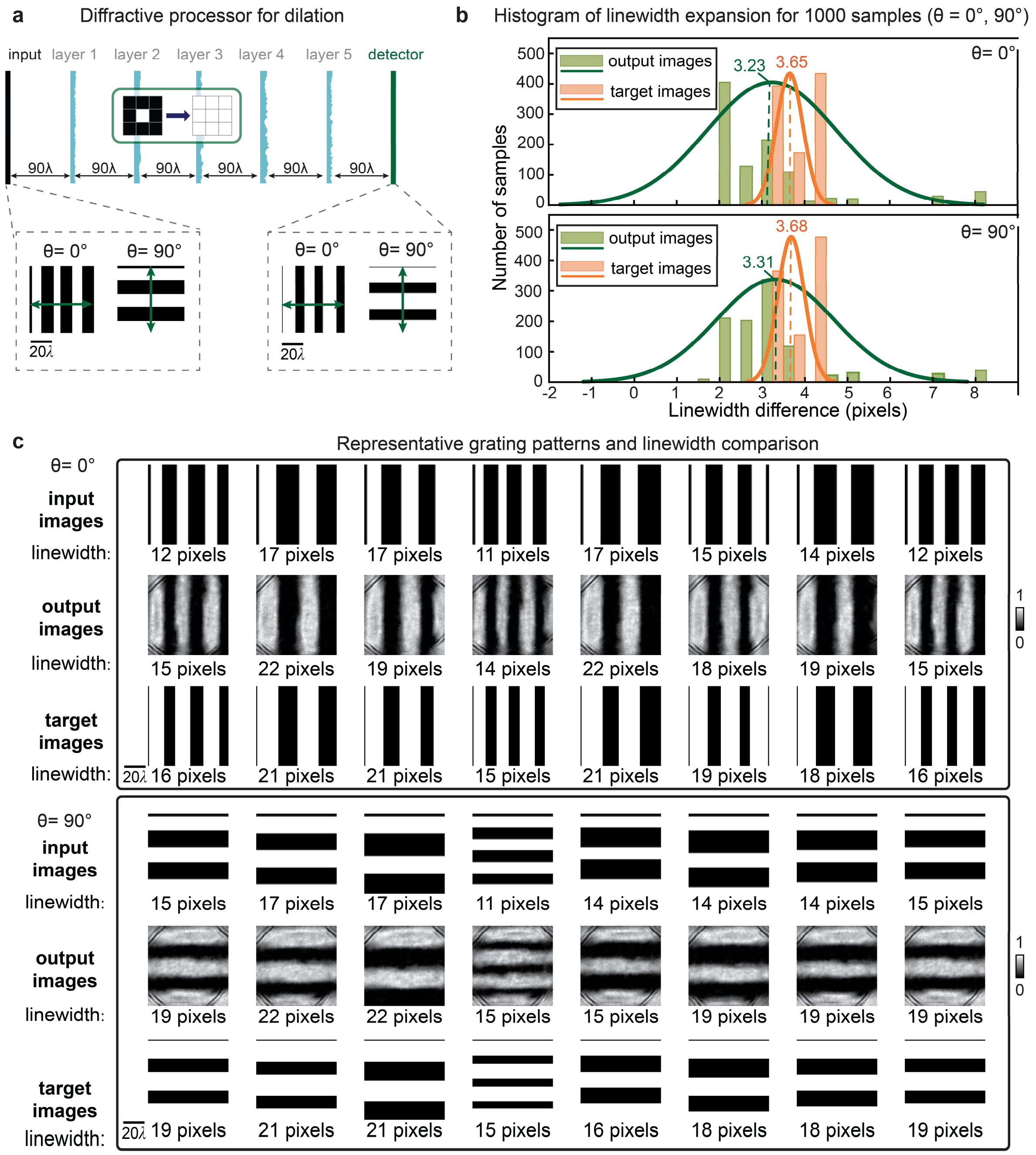

Figure S5: Quantitative evaluation of the diffractive processor on isotropic dilation tasks. (**a**) Representative examples of grating inputs, diffractive outputs, and target images for both orientations. The measured linewidths confirm that the diffractive processor performs robust and orientation-independent dilation across variable spatial frequencies and feature widths. (**b**) Histograms and fitted Gaussian curves compare linewidth differences for 1000 test samples, yielding mean expansions of 3.65 and 3.31 pixels for horizontal and vertical gratings, respectively. (**c**) Representative input, output, and target patterns demonstrate consistent, orientation-independent dilation across varying grating widths.

Cascaded diffractive processors for morphological image processing

To provide a detailed visualization of the internal phase encoding mechanisms, **Figure S6a** presents the phase distribution maps of all diffractive layers in the cascaded morphological opening processor used for denoising tasks. The system consists of two separately optimized diffractive networks—one performing erosion and the other dilation—cascaded sequentially to realize a complete morphological opening operation. Each network comprises five diffractive layers, with an inter-layer spacing of 90λ, and both are trained using a 5 × 5 all-ones structuring element to perform single-step morphological transformations. The upper row of **Figure S6b** shows the phase patterns of the erosion network (layers 1–5), while the lower row illustrates the corresponding layers of the dilation network. The color scale from 0 to 2π represents the phase modulation imparted by each diffractive neuron.

The phase maps reveal distinct yet complementary distributions across the two networks: the erosion layers exhibit concentrated and inward-propagating phase modulations corresponding to intensity contraction, whereas the dilation layers display outward-spreading features that restore expanded structures. The progressively smoother phase transitions in deeper layers indicate the network's convergence toward stable morphological representations. Together, these results visualize how the cascaded diffractive architecture physically encodes morphological erosion and dilation processes in free space, thereby achieving all-optical denoising functionality.

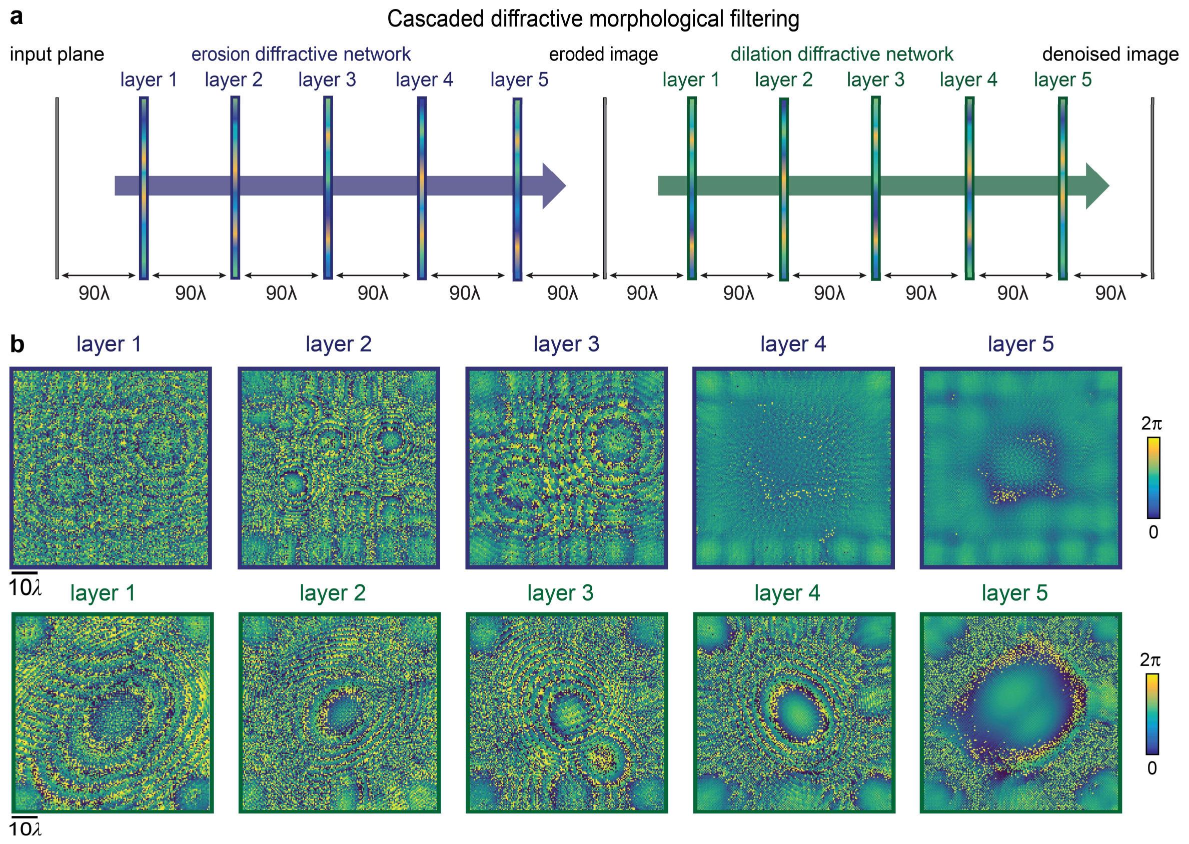

Figure S6: Phase distribution maps of erosion and dilation processors for denoising tasks. (**a**) Cascaded diffractive network for morphological opening, composed of separately optimized erosion and dilation networks. (**b**) Phase distribution maps across all diffractive layers in the cascaded morphological filtering system. Upper row: erosion processor layer 1 to layer 5; Lower row: dilation processor layer 1 to layer 5

To further examine the external generalization capability of the cascaded diffractive denoising architecture, Supplementary **Figure S7** presents detailed results obtained using real industrial inspection images from the Magnetic Tile Surface Defects dataset. A total of 50 images were randomly selected and used exclusively for testing, without any retraining or modification of the diffractive networks. The same cascaded erosion–dilation configuration used in the synthetic experiments was applied here to suppress noise patterns associated with defect formation and imaging processes.

Figure S7a presents representative qualitative results from the Magnetic Tile Surface Defects dataset. For each sample, the clean grayscale surface-defect image and its corresponding clean defect mask are shown in the leftmost column as structural references. To emulate realistic industrial inspection conditions, salt-noise perturbations with SNR = 20, 15, 10 and 5 dB were first added to the real defect images. The noisy images were then binarized to generate noisy defect masks, which contain both the structural defect region and noise-induced false responses. These noisy masks were subsequently processed by the cascaded erosion–dilation diffractive network, acting as a morphological post-processing module to suppress noise artifacts while preserving the defect geometry. Across all noise levels, the denoised outputs recover the principal defect contours and remove most isolated noise points, demonstrating that the proposed optical denoiser effectively functions as a robust refinement stage following noisy segmentation.

Figure S7b reports quantitative statistics over 200 randomly selected test samples. For the noisy segmentation masks, the initial PSNR values range from 5–20 dB depending on the SNR level of the input images. After morphological post-processing, the average PSNR increases to approximately 13.43 dB across all conditions. Likewise, the SSIM improves from ~0.50–0.60 for the noisy masks to an average of ~0.72 after diffractive denoising, indicating substantial recovery of structural consistency with respect to the clean ground-truth masks. These results show that the cascaded diffractive denoising architecture remains effective when operating as a post-segmentation refinement module on noisy real-world inspection images, confirming its robustness and external generalization beyond synthetic datasets.

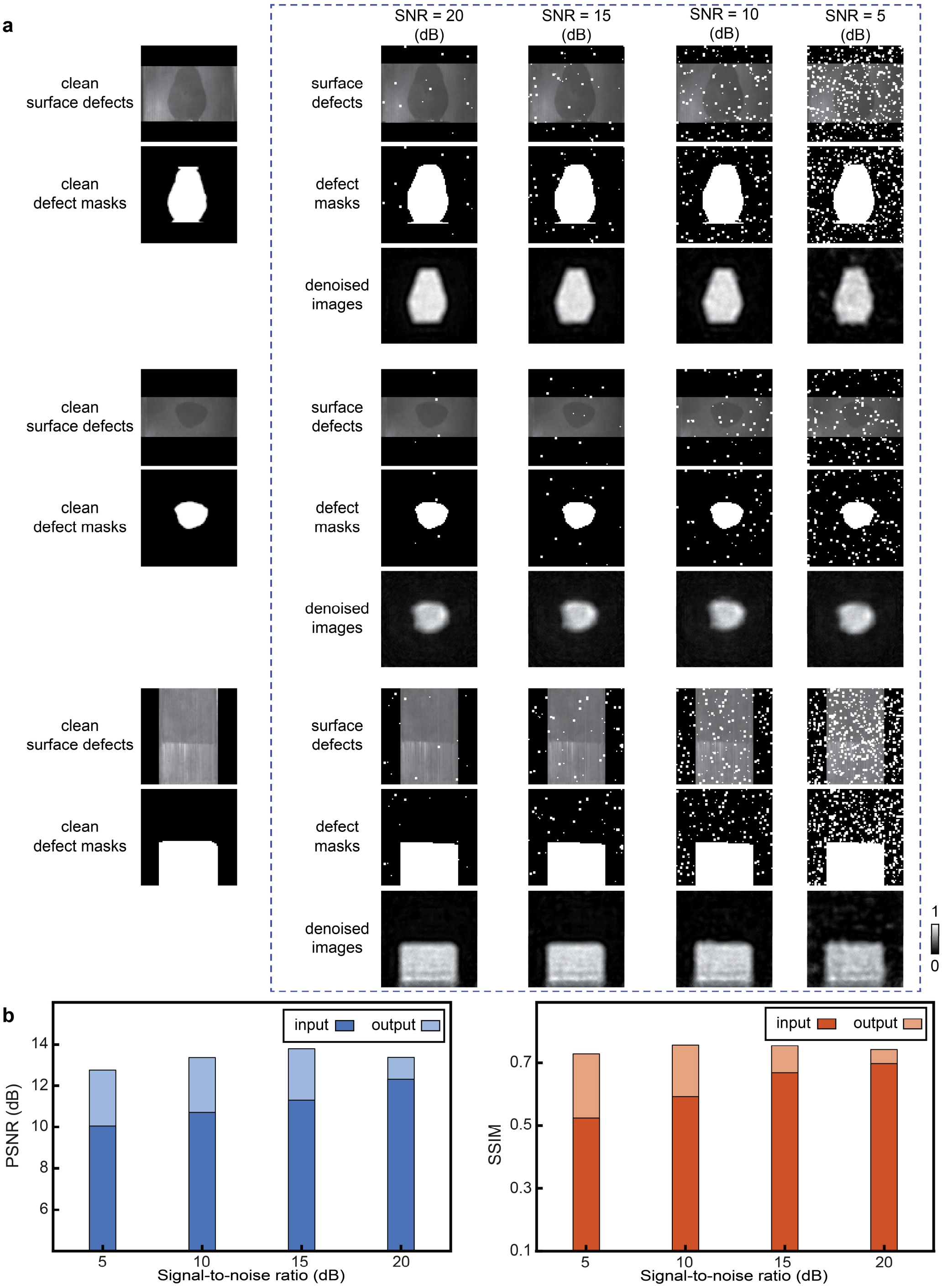

Figure S7. External generalization to real surface-defect images. (**a**) Representative results on the Magnetic Tile Surface Defects dataset: real grayscale defect images and their binary masks (left), and denoised outputs of the cascaded erosion–dilation diffractive network under different input noise levels (SNR = 20, 15, 10, 5 dB). The main defect geometry is preserved while most salt noise is suppressed. (**b**) Quantitative evaluation over 50 test images: the average PSNR increases from 5 – 20 dB for the noisy inputs to ≈13.4 dB after denoising, while the SSIM improves from about 0.5 – 0.6 to ≈0.72, indicating effective structural restoration on real industrial data.

To assess the spatial robustness of the cascaded diffractive morphological opening system, controlled lateral misalignments (Δx) were introduced between the erosion and dilation sub-networks along the x–y plane (**Figure S8a**). This test mimics realistic layer-registration errors that may occur during device fabrication or alignment. Each network pair was axially separated by 90 λ, and the lateral offset was varied from 0.75 μm to 4.5 μm (equivalent to 5–30 pixels in simulation). Representative output images from the EMNIST, Eyeglasses, and Leaf datasets are shown in **Figure S8b**, where only minor degradation is visible even under the largest offset. Quantitatively, the EMNIST dataset exhibited the highest overall stability, with SSIM values decreasing only slightly from 0.784 (Δx = 5 pixels) to 0.768 (Δx = 30 pixels), corresponding to a 2.0 % reduction. The Eyeglasses dataset showed a similar trend, from 0.716 to 0.703 (≈ 1.8 % drop), while the Leaf dataset decreased from 0.741 to 0.731 (≈ 1.3 % drop). Across all datasets, the average decline in SSIM remained below 3 %, indicating that lateral displacements up to 4.5 μm do not substantially impair reconstruction quality (**Figure S8c**).

These results demonstrate that the cascaded diffractive architecture exhibits strong spatial robustness, maintaining nearly invariant denoising performance under moderate layer misalignments. This tolerance to x–y displacement further confirms the practical manufacturability and alignment resilience of multi-stage diffractive systems for all-optical morphological computing.

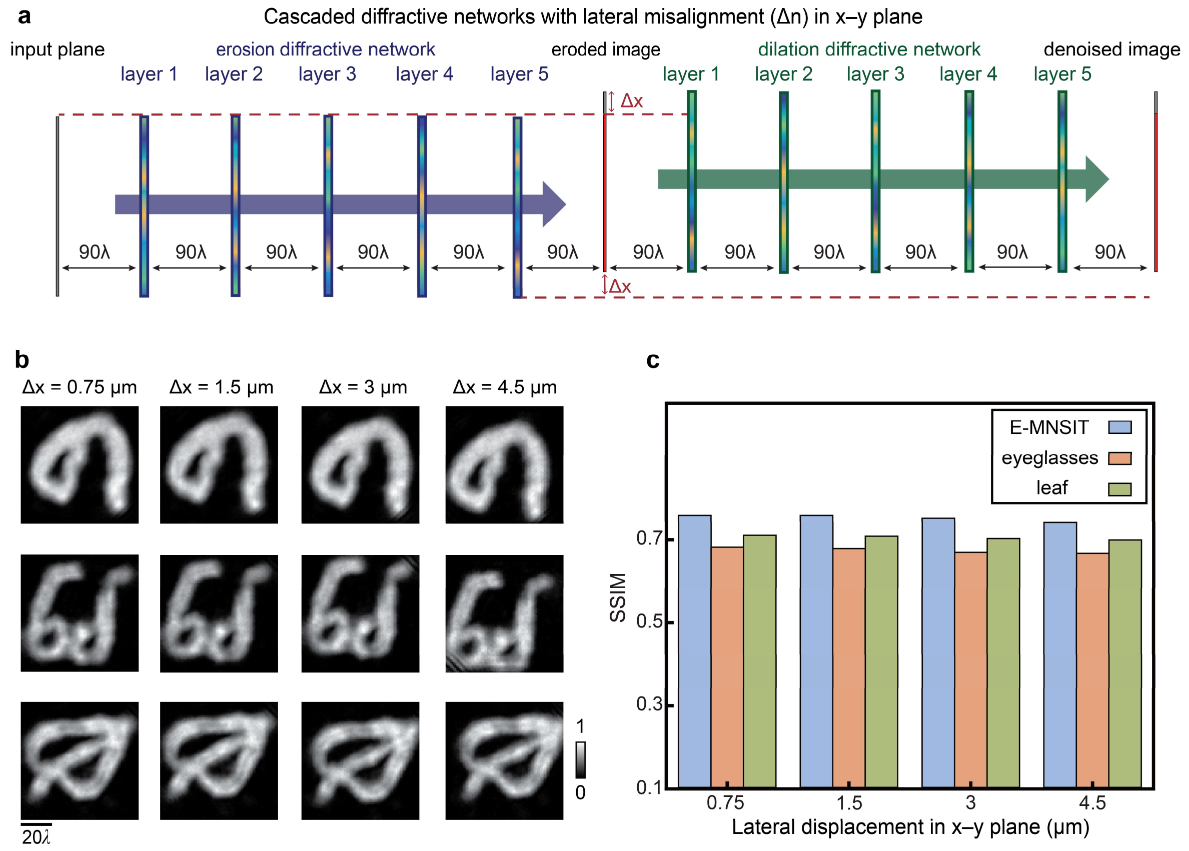

Figure S8: Robustness analysis of cascaded diffractive networks under lateral misalignment. (**a**) Schematic of the cascaded diffractive morphological opening system with an intentionally introduced lateral misalignment (Δx) between the erosion and dilation networks along the x–y plane. (**b**) Representative output images from the EMNIST, Eyeglasses, and Leaf datasets under different misalignment distances (Δx = 0.75 μm, 1.5 μm, 3 μm, and 4.5 μm). (**c**) Structural Similarity Index values of the denoised results as a function of lateral displacement.

Tuning structural-elements size within all-optical morphological transformations

Although free-space optical propagation alone can inherently induce effects resembling morphological erosion—due to diffraction-induced spreading and edge attenuation—such behaviors are typically uncontrolled, often exhibiting severe edge artifacts and unpredictable geometric distortions (see **Figure S9**). To overcome these limitations, we aim to train diffractive processors that can accurately and efficiently approximate diverse morphological transformations,

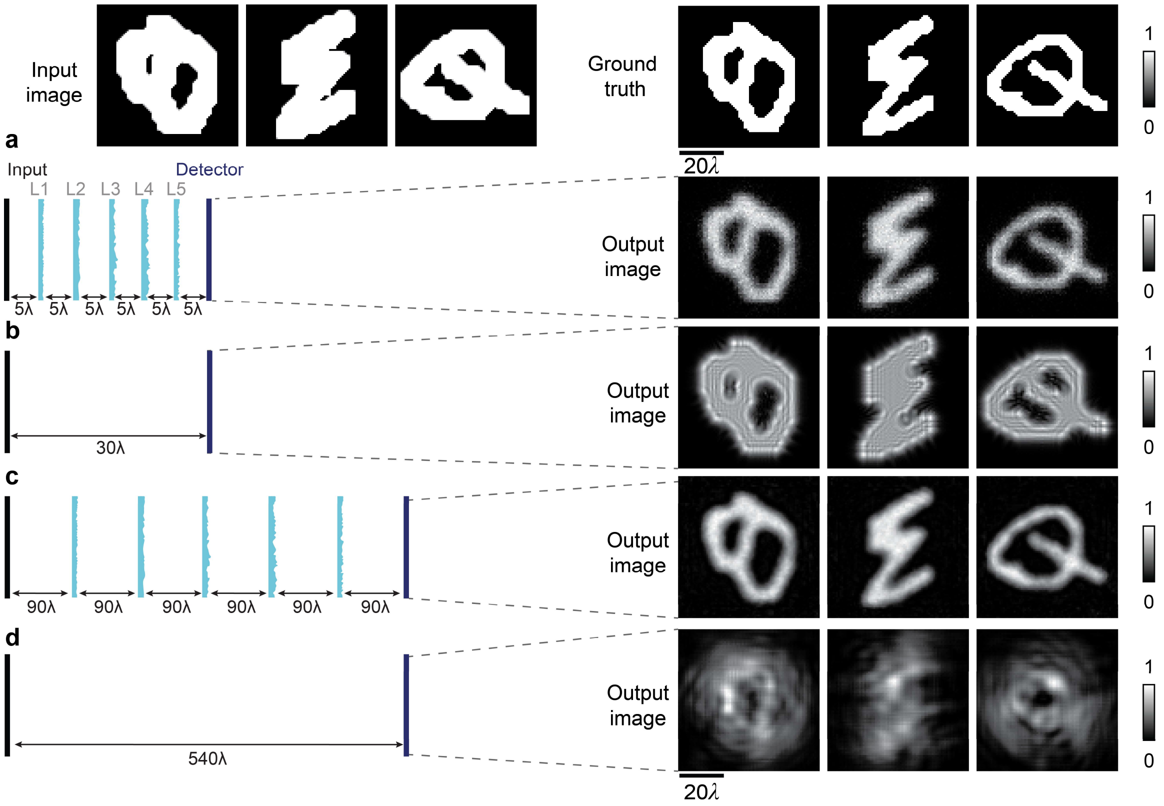

Figure S9: Comparative analysis of controlled diffractive processing versus uncontrolled free-space propagation effects in morphological erosion. (**a**) Compact diffractive processor (5λ spacing) demonstrates effective erosion performance with controlled morphological transformation. (**b**) Pure free-space propagation (30λ) generates uncontrolled diffraction-induced "erosion-like" effects characterized by severe edge artifacts and unpredictable morphological distortions. (**c**) Extended spacing diffractive processor (90λ) achieves superior performance compared to compact configurations, attributed to enhanced utilization efficiency of diffractive modulation elements. (**d**) Long-distance free-space propagation (540λ) results in complete image degradation due to excessive diffraction effects, rendering morphological features indiscernible.

enabling robust and tunable optical computation for image processing tasks. We first directly train a single diffractive optical processor to perform morphological operations corresponding to higher-order structuring elements. The results are presented in **Figure S10**. By varying the size of the structuring element S from $S_{(3\times3)}$ to $S_{(5\times5)}$, $S_{(7\times7)}$, and $S_{(9\times9)}$. Here, $S_{(n\times n)}$ denotes a square structuring element of size $n \times n$, where the values are set to all ones to represent standard isotropic morphological operations. We quantitatively evaluate the degree of erosion by measuring the pixel width reduction of periodic grating structures. The original input grating has a structural width of 17 pixels. After processing with a standard erosion optical processor, the width decreases to 15 pixels. With increasing erosion strength, the widths further reduce to 13, 10, and 9 pixels, respectively. Similarly, **Figure S10a-b** illustrates the performance of dilation processors with different degrees of expansion. The input grating structure is 15 pixels wide, which expands to 17 pixels using the standard dilation processor, and further to 18, 20, and 23 pixels with processors of increasing dilation strength. These results demonstrate that our morphological transformation toolbox can be customized to achieve varying degrees of structural change by designing different diffractive configurations. To assess image quality under different degrees of morphological transformation, we further analyze the SSIM and mean squared error (MSE) across test datasets, as shown in **Figure S10c**. For the erosion task, the SSIM remains above 0.7 when the size reduction is within 50% of the original structure. However, when the target size decreases beyond 50%, the SSIM drops significantly. For the dilation task, SSIM stays above 0.5 for size increases under 50%, but declines to around 0.2 when the change exceeds that threshold.

To evaluate the scalability of diffractive morphological processors for realizing large effective structuring elements, we adopted an iterative architecture composed of cascaded small-kernel ($S_{(3\times3)}$) modules (**Figure S11a**). Each trained diffractive network was repeatedly applied to the

output of the previous stage, physically emulating multi-round optical erosion or dilation. This recursive composition, well established in digital morphology, allows progressive transformation strength to be achieved without retraining new large-kernel models. Notably, in the current study, the cascaded results are obtained by directly stacking individually trained $S_{(3\times3)}$ modules, without cascade-aware re-optimization (i.e., neither stage-wise fine-tuning after cascading nor end-to-end joint optimization of the entire cascade).

As shown in **Figure S11b**, the baseline single-stage erosion processor achieved SSIM values of 0.925, 0.835, and 0.897 for the EMNIST, gratings, and QuickDraw-cloud datasets, respectively, while the corresponding dilation processor reached 0.825, 0.676, and 0.737. When increasing the number of cascaded iterations from 1 to 4 under this non-reoptimized setting, SSIM gradually decreases due to cumulative error propagation across stages, manifested as accumulated diffraction noise and contrast loss. For erosion, the SSIM values were 0.925, 0.888, 0.819, and 0.701 for EMNIST; 0.835, 0.752, 0.596, and 0.378 for gratings; and 0.897, 0.834, 0.730, and 0.579 for QuickDraw-cloud. For dilation, a similar trend was observed, with SSIMs of 0.825, 0.714, 0.619, and 0.537 (EMNIST), 0.676, 0.524, 0.399, and 0.297 (gratings), and 0.737, 0.606, 0.503, and 0.423 (QuickDraw-cloud). Moreover, we note that cascade-aware re-optimization—such as stage-wise fine-tuning using the actual outputs of preceding stages or end-to-end joint training with a global loss on the final output—is expected to mitigate cumulative-error effects and further improve the performance of deeper cascades.

Despite this moderate quality degradation, the effective transformation depth increased substantially with each additional cascade, confirming that the iterative diffractive scheme can flexibly emulate larger structuring elements while maintaining SSIM > 0.7 for up to two iterations on most datasets. As shown in **Figure S11c**, the erosion and dilation effects become progressively

stronger with iteration number, yet the overall image fidelity remains acceptable, validating the robustness of the cascaded optical morphology operator.

Besides isotropic morphological transformations, directional erosion and dilation operations can also play a key role in edge enhancement to image denoising that require selective extraction of features with certain orientations. In this work, we design a series of all-optical morphological transformation processors based on diverse structural elements. **Figure S12** illustrates the variety of these processors. We employ structural elements in the form of all-1 matrices, including 1×3 and 3×1 configurations, to perform image erosion and dilation along the horizontal and vertical directions, respectively. Using input test sets similar to those in Section 2.1, we evaluate the performance of these processors for directional erosion tasks. The results show that, despite changes in the structural elements, the diffractive networks can be effectively trained to match the performance of electronic morphological networks. For the EMNIST and several unseen QuickDraw test sets, the average SSIM of the output images reaches approximately 0.803 for the erosion task. Similarly, for the dilation task, the diffractive networks also demonstrate strong adaptability under different structural configurations, achieving an average SSIM of 0.61 on the same test sets.

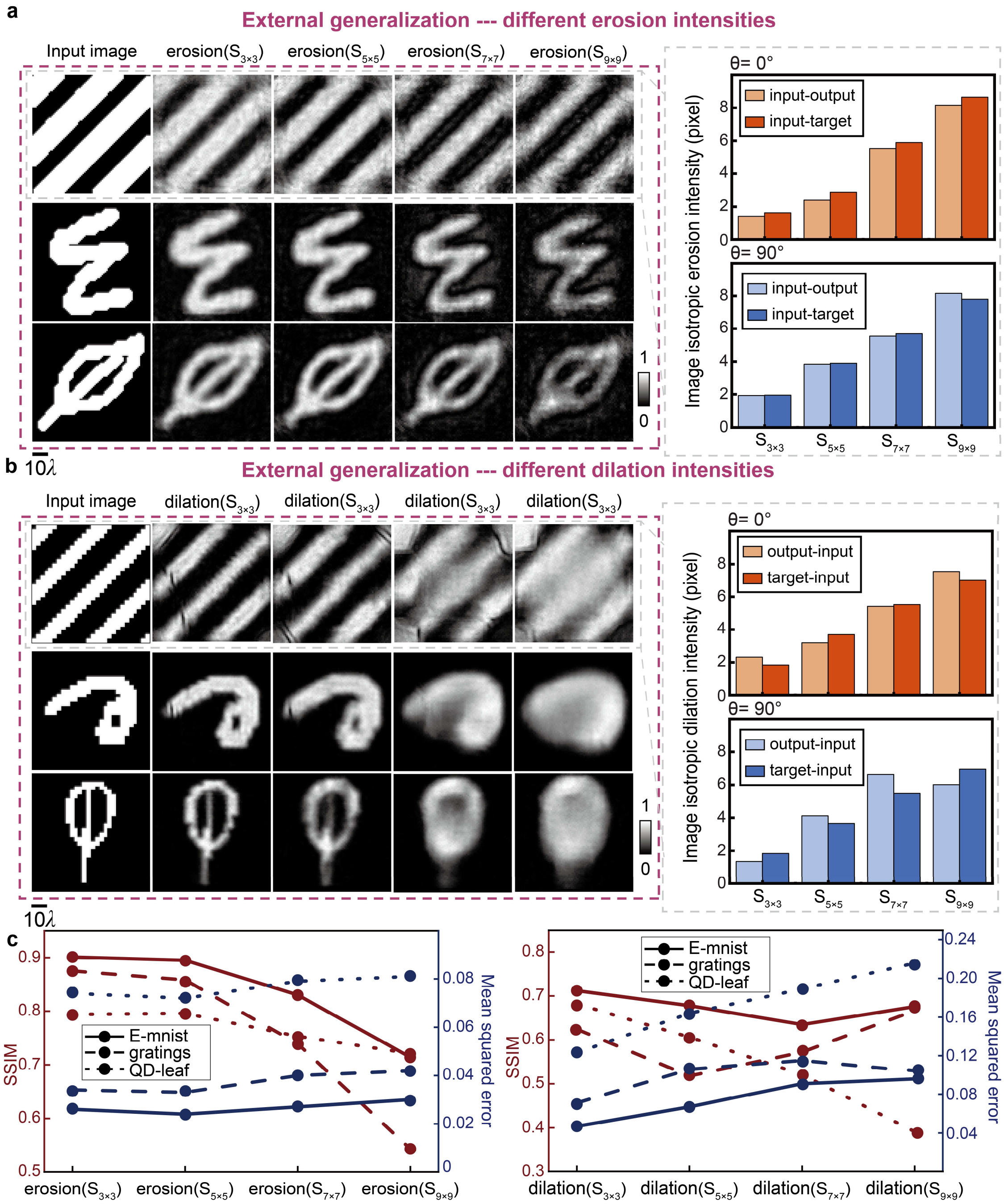

Figure S10. Generalization of all-optical processors to tasks with different degrees of morphological variation. (**a**) Quantitative analysis of changes in image structure size under different erosion levels. (**b**) Quantitative analysis of changes in image structure size under different dilation levels. (**c**) Analysis of image quality after different degrees of image morphological changes by optical processors.

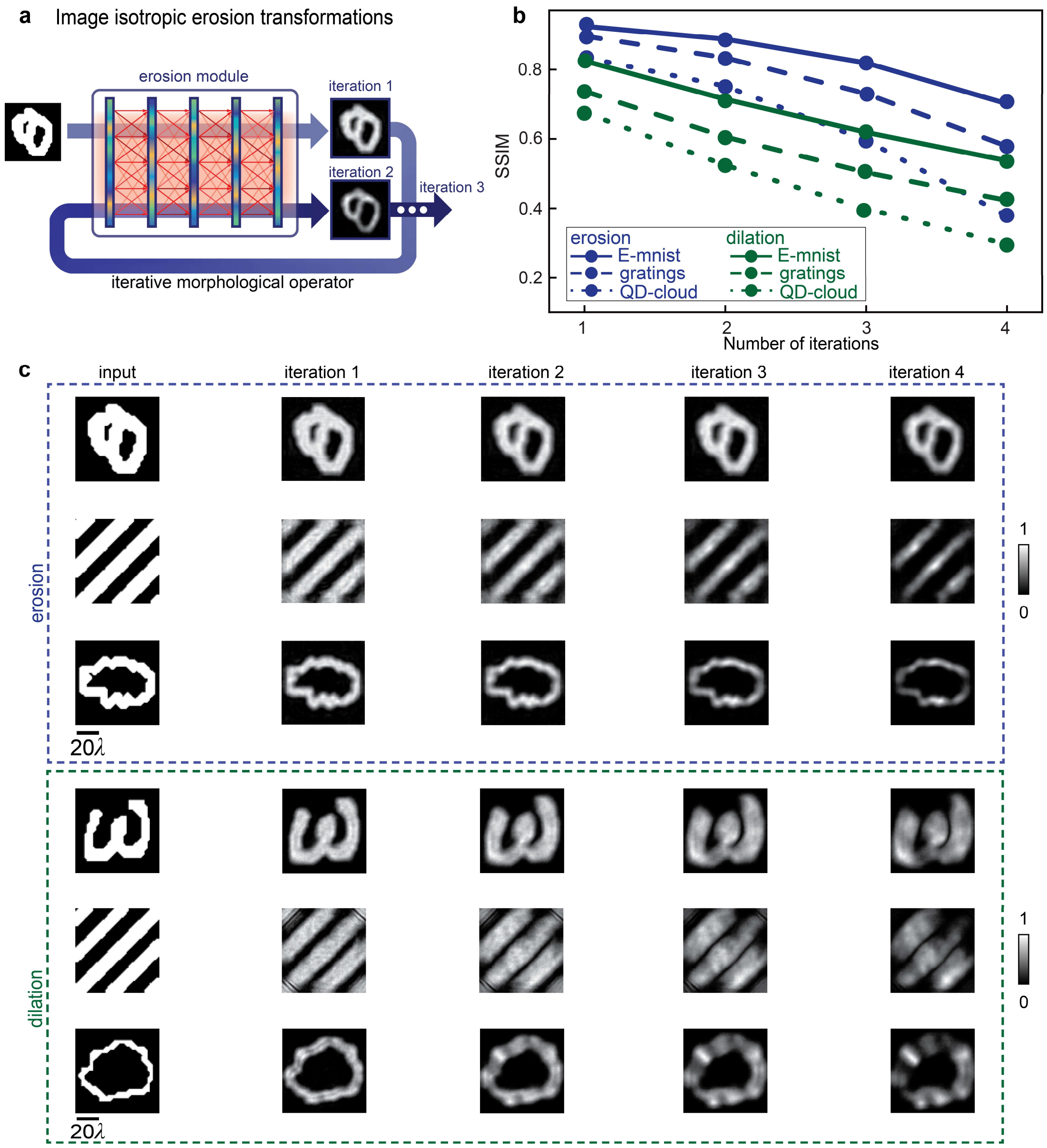

Figure S11. Iterative and cascaded diffractive morphological transformations. (**a**) Schematic of the iterative diffractive morphology operator, where the same trained erosion or dilation module is reused for multiple iterations to achieve progressively stronger morphological effects. (**b**) Structural Similarity Index performance of diffractive erosion and dilation models on the EMNIST, gratings, and QD-Cloud datasets as a function of the number of iterations. (**c**) Representative images showing the evolution of erosion (top) and dilation (bottom) results over successive iterations for different input patterns.

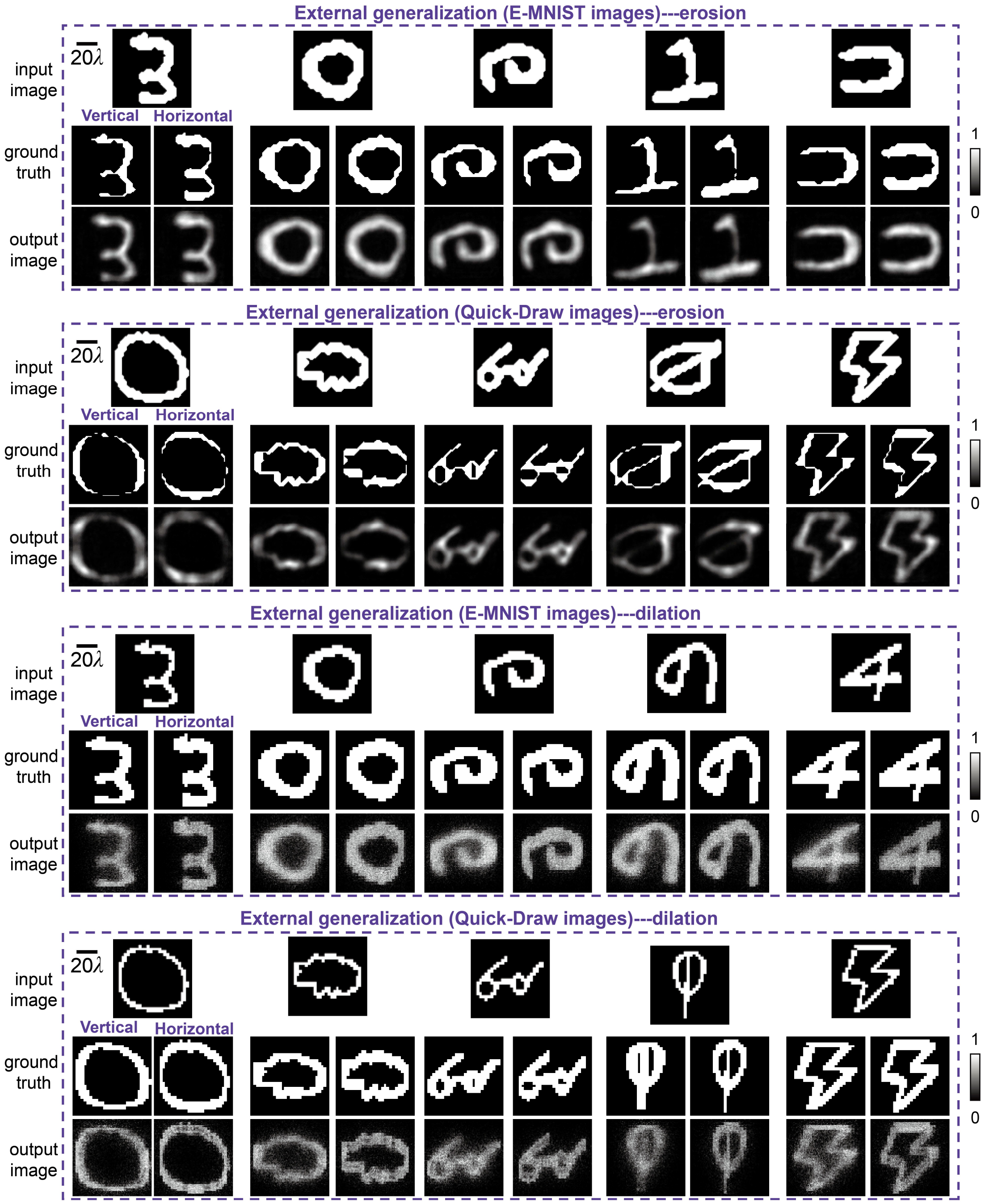

Figure S12. All-optical morphological transformations of anisotropic structuring elements.

Scalability of the diffractive processor for processing large, high-resolution images

To evaluate the scalability and optical coupling characteristics of the diffractive morphological processor, we systematically analyzed its performance under varying inter-layer propagation distances (d = 5 λ , 10λ, 15λ, 20λ, 50λ, 100λ) (**Figure S13**). The diffractive networks were trained on 640 × 640 pixel inputs using stitched composite datasets and subsequently tested on unseen large-scale samples to assess generalization. The results in **Figure S13b** show that as the layer-to-layer spacing increases, the morphological fidelity measured by the SSIM gradually decreases. For the EMNIST dataset, the SSIM values were 0.721, 0.716, 0.705, 0.755, 0.491, and 0.528 for d = 5λ, 10λ, 15λ, 20λ, 50λ, 100λ, respectively, indicating the highest accuracy at d = 20λ. The gratings dataset exhibited lower overall fidelity due to its high spatial frequencies, with SSIM values of 0.536, 0.473, 0.414, 0.472, 0.202, and 0.193 under the same spacings, showing a clear degradation when d⩾50λ. For the QuickDraw–Leaf dataset, SSIM values were 0.670, 0.689, 0.675, 0.727, 0.482, and 0.513, also peaking near d = 20λ. Representative results in **Figure S13d** demonstrate that shorter inter-layer distances (5λ - 20λ) maintain sharper morphological boundaries, while excessively long separations (>50λ) reduce effective optical coupling and lead to blurring and contrast loss. These observations confirm that optimal inter-layer spacing lies around 5-20λ, balancing sufficient diffraction-based interaction and stable light-field propagation for large-scale diffractive morphological computing.

To further examine the scalability and optical coupling behavior of the diffractive morphological processors at larger spatial dimensions, we extended the input resolution to 1280×1280 pixels and evaluated performance under varying inter-layer propagation distances (**Figure S14a**). As shown in **Figure S14b**, the morphological fidelity, quantified by the (SSIM), decreased with increasing propagation distance due to weakened diffractive coupling between

layers. Quantitatively, the SSIM values for the EMNIST, gratings, and QuickDraw-Leaf datasets were 0.656, 0.483, 0.634 at d = 5λ; 0.419, 0.134, 0.405 at d = 50λ; 0.431, 0.126, 0.423 at d = 100λ; and 0.427, 0.104, 0.416 at d = 200λ, respectively. These results indicate that shorter layer-to-layer separations (~5λ) ensure stronger optical interference and higher morphological accuracy, whereas excessively long propagation paths (≥100λ) lead to significant quality degradation. Representative phase profiles of the trained diffractive layers for d = 5λ are presented in **Figure S14c**, showing uniformly distributed phase modulation across the large-area aperture, validating stable optimization at this scale. The reconstructed examples in **Figure S14d** further confirm that dense layer spacing preserves clear morphological boundaries across datasets, while larger spacing causes blurring and loss of fine structural details. Overall, these findings highlight that maintaining compact optical coupling is essential for achieving high-fidelity morphological transformations in large-scale diffractive processors.

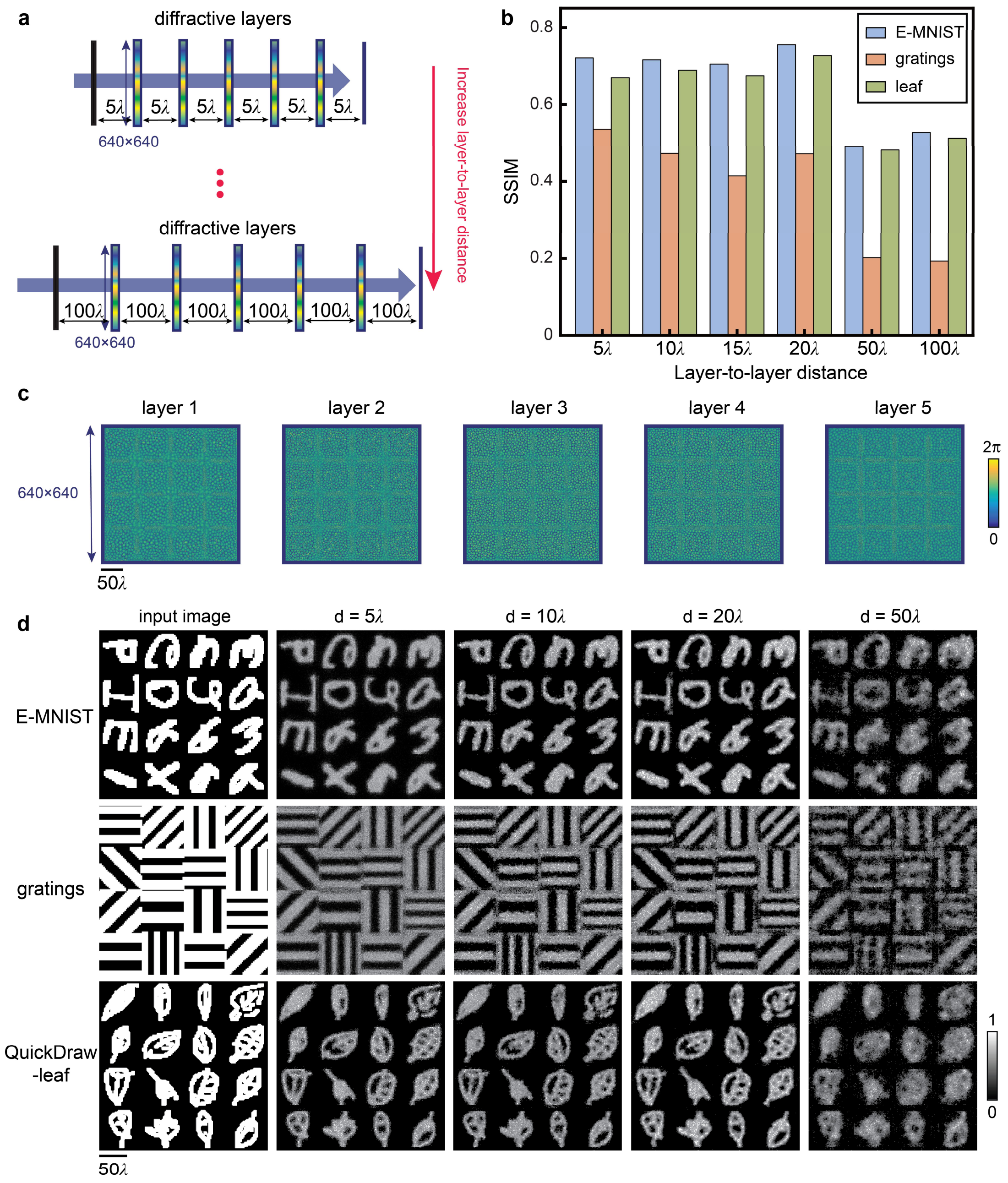

Figure S13. Scalability and inter-layer spacing optimization of diffractive morphological processors. (**a**) Schematic of diffractive processor configurations with varying inter-layer distances (d = 5λ - 100λ) for large-scale image processing. (**b**) Structural similarity (SSIM) performance across E-MNIST, gratings, and Leaf datasets as a function of inter-layer spacing, showing that shorter propagation distances (around 5λ - 20λ) yield higher fidelity due to stronger layer coupling, whereas excessively large separations reduce effective optical interaction and degrade image quality. (**c**) Representative phase maps of the trained diffractive layers(640×640) for d = 5λ. (**d**) Output examples from EMNIST, gratings, and QuickDraw-Leaf datasets under different layer spacings.

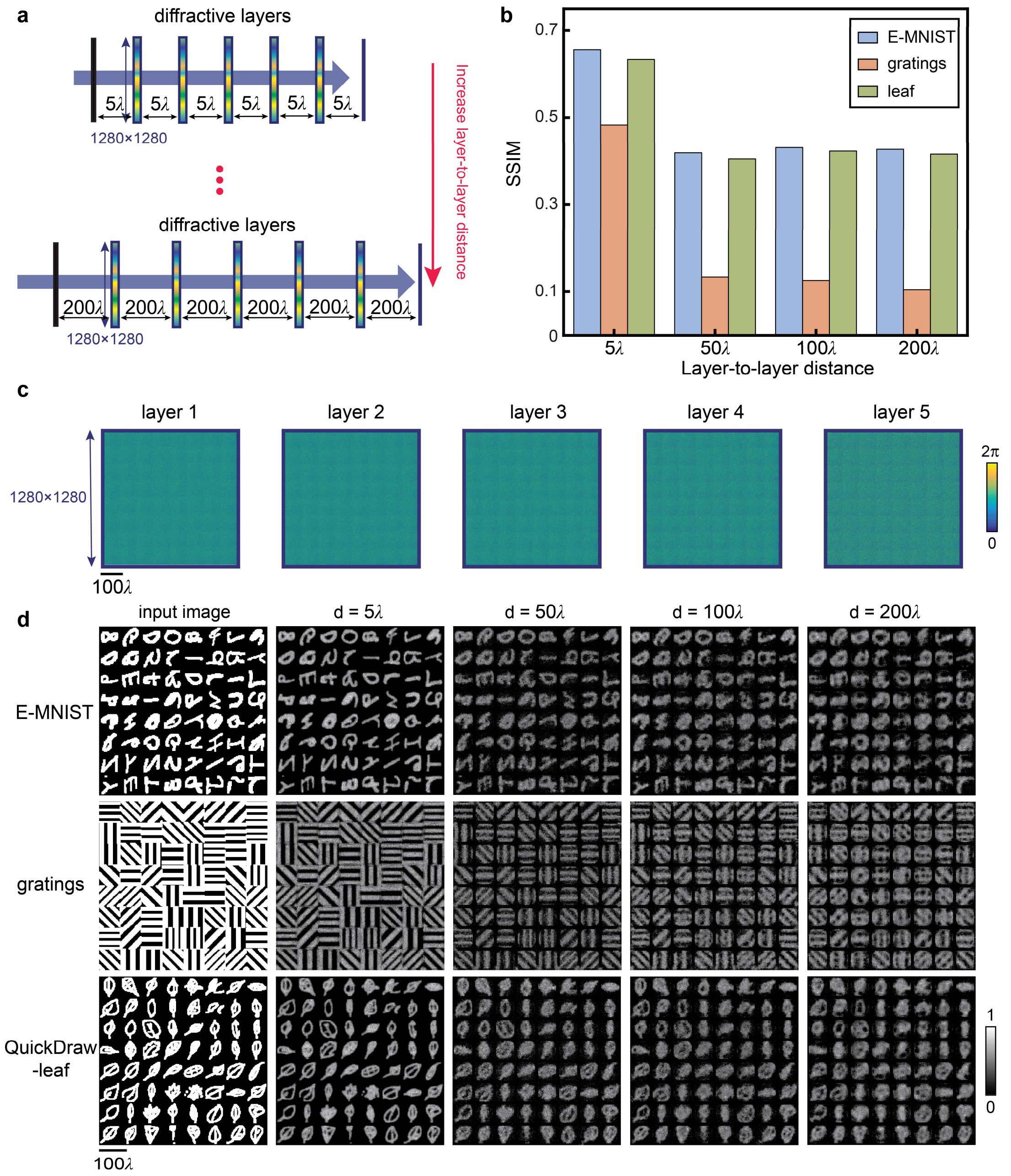

Figure S14. Scalability of diffractive morphological processors for large-scale (1280 × 1280 pixels) image inputs and inter-layer spacing optimization. (**a**) Schematics of diffractive processor configurations with varying inter-layer spacings (d = 5λ - 200λ) designed to evaluate optical coupling in large-area processors. (**b**) Structural similarity (SSIM) performance across EMNIST, gratings, and QuickDraw-Leaf datasets as a function of inter-layer distance. The results reveal that shorter propagation distances(d = 5λ) provide stronger diffractive coupling and higher morphological fidelity, whereas excessively large separations(d≥100λ) substantially degrade the output quality. (**c**) Representative phase maps of the trained diffractive layers(1280×1280) for d = 5λ. (**d**) Output examples from EMNIST, gratings, and QuickDraw-Leaf datasets under different layer spacings.

Figure S15 presents the numerical and experimental results of all-optical erosion using amplitude-encoded EMNIST inputs on the reflective SLM platform. Prior to the experiment, the diffractive network was numerically evaluated under a diffraction-efficiency constraint of 20%. Under this condition, the simulated structural-similarity index (SSIM) for the EMNIST test set reached approximately 0.88, indicating that the network preserved good transformation fidelity while suppressing background leakage in the amplitude domain. However, during the experimental validation, the output quality deteriorated noticeably at the same 20% diffraction-efficiency setting. This degradation was primarily caused by the limited optical power of the supercontinuum light source at $\lambda = 635$ nm, which resulted in an insufficient signal-to-noise ratio at the sensor plane. To compensate for this limitation, we increased the diffraction-efficiency threshold in the loss term to 50% and retrained the network. Under this condition, the simulated SSIM slightly decreased to ≈ 0.80, reflecting a moderate trade-off between diffraction efficiency and morphological-transformation fidelity.

With the higher diffraction-efficiency setting, the experimental results exhibited clearly visible output images, confirming effective erosion behavior on most letter strokes. At the same time, due to the stronger energy concentration in the diffracted field, local spot spreading was observed in certain spatial regions of the output images, which led to weakened erosion strength in some fine structures. This phenomenon is consistent with the expected propagation-induced broadening at high diffraction-efficiency regimes in the reflective geometry. These observations provide useful guidance for future device optimization and indicate that the balance between diffraction efficiency and morphological fidelity will be an important design parameter for practical integrated implementations.

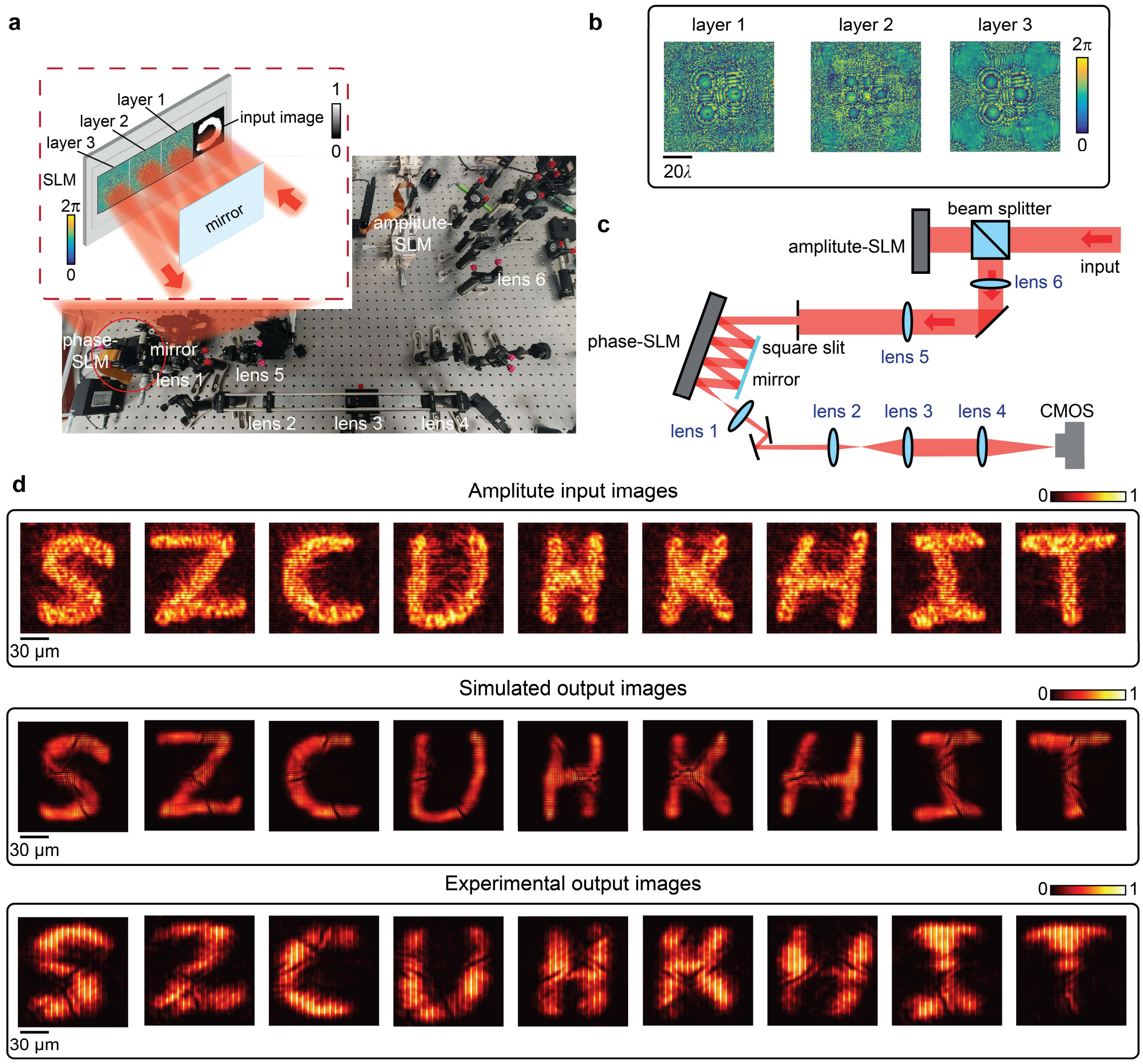

Figure S15. Experimental demonstration of diffractive morphological erosion on amplitude-encoded images. (**a**) Photo and scheme showing the reflective phase-SLM platform emulating a multi-layer diffractive network using mirror reflections. (**b**) Optimized phase distributions of the three diffractive layers used for the erosion task. (**c**) Experimental setup to characterize the reflective-mode diffractive network for amplitude objects, where a collimated laser beam is first modulated by an amplitude SLM to encode the input letter images and then sent to the phase-only SLM–mirror assembly for diffractive processing before being relayed and imaged onto a CMOS camera. (**d**) Representative results for erosion of amplitude images: amplitude input images (top), numerically simulated output images (middle), and experimentally captured output images (bottom).
